# Developing a Conceptual Framework for an Age-Friendly Health System: A Scoping Review

**DOI:** 10.34172/ijhpm.2023.7342

**Published:** 2023-06-07

**Authors:** Badrye Karami, Abbas Ostad-Taghizadeh, Arash Rashidian, Maryam Tajvar

**Affiliations:** ^1^Department of Health Management and Economics, School of Public Health, Tehran University of Medical Sciences, Tehran, Iran; ^2^Department of Disaster & Emergency Health, School of Public Health, Tehran University of Medical Sciences, Tehran, Iran

**Keywords:** Age Friendly, Health System, Conceptual Framework, Scoping Review

## Abstract

**Background:** Population aging is usually associated with increased health care needs. Developing an age-friendly health system with special features, structure, and functions to meet the special needs of older people and improving their health status and quality of life is essential. This study aimed to develop a conceptual framework for an age-friendly health system, which would offer a conceptual basis for providing the best possible care for older people in health system to let them experience a successful, healthy, and active aging.

**Methods:** A scoping review was used to design the conceptual framework based on Arksey and O’Malley’s model, including six stages, with the final stage of using expert’s opinions to improve and validate the initial framework. The health system model of Van Olmen, was selected as the baseline model for this framework. Then, by reviewing the available evidence, the characteristics of an age-friendly health system were extracted and incorporated in the baseline mode.

**Results:** Using the electronic searching, initially 12 316 documents were identified, of which 140 studies were selected and included in this review study. The relevant data were extracted from the 140 studies by two reviewers independently. Most studies were conducted in 2016-2020, and mostly were from United States (33.6%). To have an age-friendly health system, interventions and changes should be performed in functions, components and objectives of health systems. This system aims to provide evidence-based care through trained workforces and involves older people and their families in health policy-makings. Its consequences include better health acre for older people, with fewer healthcare-related harms, greater care satisfaction and increased use of cost-effective health services.

**Conclusion:** To meet the needs of older people, health systems should make interventions in their functions for better performance. In line with these changes, other parts of society should work in harmony and set the health of older people as a top priority to ensure they can have a successful aging.

## Background

 Population aging, which refers to an increase in the ratio of people over 60 years to the total population, is a global phenomenon that stems from improved living standards, health, socio-economic conditions, and the increasing life expectancy as well as the implementation of birth control policies. According to World Population Prospects 2019, the world’s population over the age of 65 will be more than double between 2019 and 2050; by the year 2050, 1 in 6 people in the world will be over the age of 65, a noteworthy increase from 1 in 11 in 2019.^1^

 The phenomenon of population aging affects various social, economic, and political aspects in countries, and significantly increases the costs of health, social services, and welfare in society.^2,3^ Sometimes the cost of treatment in the last two years of life equals the cost of the rest of life.^4^ Since older people often have complex health and social needs and experience several chronic diseases, the health system is one of the places with which the older people have the most contact after their home and neighborhood.^5^ According to the definition of the World Health Organization (WHO), health systems include all organizations, institutions, and resources that are established for the production of health actions and improving health status.^6,7^ Therefore, to meet the health needs of older people and promote their health status, the health system should be as friendly as possible to older people^5^ and must make considerable efforts to maximize their ability to live an active life and delay their disability.^8^ Likewise, the health system should take special measures to improve insurance coverage, services for older people, more attention to older people with low socioeconomic status, expansion of outpatient services for them, and using trained workforces for older people.^9^

 Age-friendly health systems are defined as health systems in which older people receive the best possible care, with fewer healthcare-related harms, greater care satisfaction, and optimized value.^10^ “[This system] would keep older adults healthy, be proactive in addressing potential health needs, prevent avoidable harms, improve healthcare for those with serious illnesses who need end-of-life care, and support family caregivers throughout.”^11^ This system aims to provide evidence-based care through trained workforces along with a wide range of community-based services and to engage older people and their families in the care process, thus providing better health service with cost-effective use of resources and reduction of devaluation.^10^

 Although there are different definitions for an age-friendly health system and despite the existence of other similar models eg, structural framework model^10^ and “the 4Ms” model,^12^ no specific conceptual framework can characterize the dimensions and indicators of a health system. Therefore, designing a conceptual framework for an age-friendly health system is necessary as an instrument for evaluating different health systems and identifying areas with weaknesses that can be improved so as to pave the way for a more age-friendly health system. In this study, the aim was to develop a conceptual framework for an age-friendly health system using a scoping review, with the ultimate goal of providing better health services for older people to help them experience successful, healthy, and active aging.

## Methods

 In this study, the conceptual framework for an age-friendly health system was designed based on the six stages of Arksey and O’Malley’s model of scoping reviews.^13^ These stages are explained as follows:

###  First Step: Identification of Research Questions

 We used the population, concept, and context (PCC) framework to refine the primary research question and purpose consists of three main components. In the case of the present research, “population” includes studies that assess each dimension of the age-friendly health system, “concept” includes the dimensions and components of the conceptual framework of an age-friendly health system and the relationship between them, and “context” includes studies with universal applicability to all health systems.

 Three main research questions were designed: What is the appropriate basic model among the existing health system models for the purpose of this study? What are the main dimensions of an age-friendly health system and the relationship between these dimensions? What are the main components of the dimensions of an age-friendly health system?

###  Second Step: The Identification of Relevant Literature

 Based on the research questions mentioned above, this step consisted of two phases:

*Phase I:* review all existing health system models and frameworks to select a basic health system model. Since designing an age-friendly health system is based on the existing health system and is not a separate concept, MT and BK examined the existing health system models and frameworks to select an optimum model that should be usable for anyone who intends to analyze or strengthen the health system. To that end, after reviewing 49 models and frameworks (eg,^14-16^), the research team agreed upon using Van Olmen’s model^17^ as the basic model. Van Olmen’s model was developed for the analysis of any health system at national, intermediate, and local levels and is applicable and standardized in contexts with special principles and values. In this model, 10 elements or functions are identified as the building blocks of any health system: (1) goals and outcomes, (2) values and principles, (3) service delivery, (4) population, (5) context, (6) leadership and governance, and (7-10) organization of resources (finances, human resources, infrastructure, supplies, knowledge, and information). This model emphasizes that a health system should move toward outcomes and goals and should be based on values and principles. In this model, there are resources as input to the health system, but the organization and provision of health services is the main element. In addition, the health system interacts with the population and other actors in a specific context.

 Van Olmen’s model is consistent with viewing the health system as a complex system and shows that the elements are interdependent. There are a large number of possible interactions in all directions between components and dimensions, such as feedback loops and production processes. The processes in this system are often nonlinear and result from forces acting between dynamic equilibria. In addition, health systems are open systems and are influenced by context and history. This is the most basic form of framework that can be applied to the systematic analysis of situations at different levels (national, regional, and health organizations) or for specific problems. This framework explains how to describe the context of each element and its relationship with other elements so that the framework can be used. Thus, one can use this framework to present the perspective of strengthening the health system based on values and principles, and this is a normative application of framework.^17^

*Phase II:* This phase includes a systematic scoping review to determine the most important dimensions and components of an age-friendly health system and the interrelationship between the dimensions. In the second phase, a scoping literature review was conducted to scope published studies and design the most appropriate conceptual framework for an age-friendly health system and its main dimensions and components. In phase II, the aim is to comprehensively examine the underlying concepts of a research area and the major sources and types of evidence available. To perform this review in the present study, after determining the research questions, a systematic search was conducted to obtain relevant scientific literature using national and international databases. The selected important and relevant international websites were searched without any time limit through a systematic search based on Persian keywords and their English equivalents with all possible combinations of important, original, and sensitive words until January 4, 2021.

###  Third Step: The Selection of Relevant Studies for Review 

**Figure 1:**
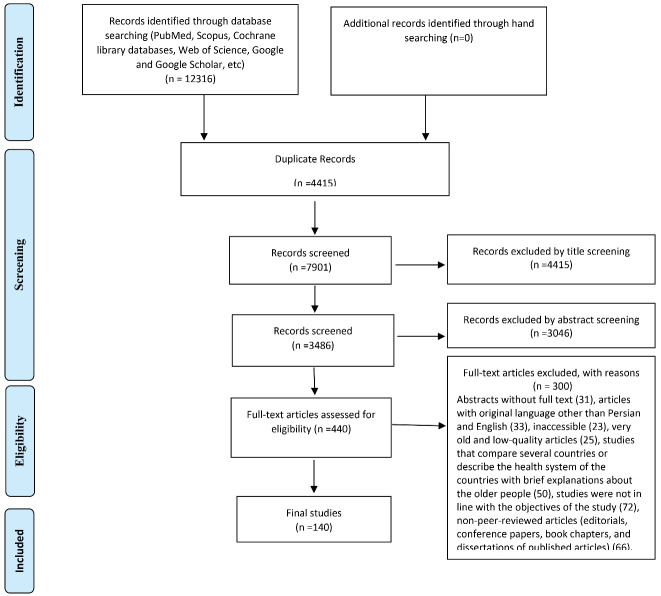
Flow Diagram Illustrating Article Selection and Elimination.

 The selection of studies were conducted based on the inclusion criteria (PCC framework), as mentioned earlier. In study selection, those with languages other than Persian or English, meeting abstracts, commentaries and letters to editor were restricted. Figure 1 presents additional information on the excluded documents. To find relevant gray literature, including various types of government reports and documents, and theses, the websites of WHO, the United Nations (UN), National Institute on Aging, and conference proceedings were searched. The same criteria, as mentioned above, were considered in a further effort for manual searching of other sources, such as the bibliography of the included papers, theses, and research projects (Table 1). However, no relevant documents were finally included in the gray literature.

**Table 1:** The Search Strategy Adopted to Search the Selected Databases

**Searching**	**Sources **	**Database/Journals**	**Keywords and Mesh**
Electronic searching	PubMed	Database	(((((((((((("age-friendly health system"[Title/Abstract]) OR ("older friendly health system"[Title/Abstract])) OR ("elderly-friendly health system"[Title/Abstract])) OR ("geriatric friendly health system"[Title/Abstract])) OR ("senior-friendly health system"[Title/Abstract])) AND (tool[Title/Abstract])) OR (plan[Title/Abstract])) OR (model[Title/Abstract])) OR ("conceptual framework"[Title/Abstract])) OR (strategy[Title/Abstract])) OR (indicator[Title/Abstract])) OR (index[Title/Abstract])) OR (experience [Title/Abstract])
	Scopus	Database	TITLE-ABS (Age-friendly health system) OR TITLE-ABS (Older friendly health system) OR TITLE-ABS (Elderly friendly health system) OR TITLE-ABS (Geriatric friendly health system) OR TITLE-ABS (Senior friendly health system) AND TITLE-ABS(Tool) OR TITLE-ABS(Plan) OR TITLE-ABS(Model) OR TITLE-ABS (Conceptual framework) OR TITLE-ABS(Strategy) OR TITLE-ABS(Indicator) OR TITLE-ABS(Index) OR TITLE-ABS(Experience)
	Web of Science	Database	
	Google Scholar	Search engine	All in the title: "Age-friendly health system" OR "Older friendly health system" OR "Elderly friendly health system" OR "Geriatric friendly health system" OR "Senior-friendly health system"
	Cochrane library	Database	Age-friendly health system*
	SID	Electronic Journal	- Translation of “Age-friendly health system” and its various synonyms in Farsi - All studies about age-friendly health system in English language
	Iranian journal of aging: Salmand	Electronic Journal	
	Journal of Gerontology	Electronic Journal	
	Journal of Caspian Health and Aging	Electronic Journal	
	Journal of Geriatric Nursing	Electronic Journal	
	The Elderly Health Journal	Electronic Journal	
	Journal of Psychology of Aging	Electronic Journal	
	Journal of Health System Research	Electronic Journal	
	Health Systems Research Journal; Hakim	Electronic Journal	
Manual searching of gray literature	Google	Search engine	
	WHO	Website	
	UN		
	National institute on aging		
	Conference proceedings		
	Bibliography of the included papers	-	
	Thesis	-	
	Research projects	-	

Abbreviations: WHO, World Health Organization; UN, United Nations.

 As shown in Figure 1, the flow diagram shows the process of identifying, reviewing, and selecting articles. First, 12 316 articles were obtained through electronic and hand searching. The duplicate records (n = 4415) were deleted before the title and abstract screening process and 7900 records remained for further review. The titles and abstracts of the remaining papers and unrelated articles were then reviewed. In all stages of screening the documents, two reviewers independently checked the citations based on the agreed inclusion and exclusion criteria in the protocol. Disagreements were resolved based on the third person’s final judgment.

 Finally, 440 articles were obtained for the full-text review to be assessed for eligibility. Finally, 140 articles met the inclusion criteria. The included studies were published between 1981 to 2020, and a significant number of them were conducted between 2016 to 2020. In addition, most published articles were conducted in the United States (33.6%).

###  Fourth Step: Charting the Data 

 Data extraction included specific details about the bibliographical information (ie, authors, title, journal, and year of publication), study design, and main findings of reviewed studies based on the basic conceptual framework’s dimensions. This process was done by using a purposefully designed data extraction form by BK and MT, developed initially based on Van Olmen’s model, and then based on the opinions of experts in the sixth stage of Arksey and O’Malley’s model,^18^ the names and features of some dimensions and components were modified, and other dimensions were added and this form was modified and completed (The final form is available in Supplementary file 1, Table S1).

###  Fifth Step: Collecting, Summarizing, and Reporting Findings 

 The included studies were developed for different objectives, used a variety of measures and methods, and included different study designs. Therefore, the results of similar dimensions or aspects of an age-friendly health system were identified and grouped. In the next stage, the findings were then reported, compared, and descriptively examined. To that end, a checklist containing the main dimensions of Van Olmen’s model was designed and the findings of the review, which corresponded to each of those dimensions, were entered into the checklist (Supplementary file 1, Table S1). In addition, other dimensions and components, which, according to the findings, should be present in a conceptual framework of an age-friendly health system but were incomplete or absent in the basic model, were included in the checklist. Then, the research team agreed to make some primary changes to the basic model. All the features obtained from performing the scoping review were then introduced to the basic model andto design the initial conceptual framework.

###  Sixth Stage: Consultation With Experts

 To complete and confirm the conceptual framework draft designed in previous steps, some of the dimensions, components, relationships, and features in the conceptual framework were modified based on the opinions of experts, and the final conceptual framework was completed and approved. To perform this step, 12 experts were selected by purposive sampling. These experts were selected from a wide range of scientific and executive experts working with the Ministry of Health who were well aware of age-friendly health systems and were selected from throughout Iran. The inclusion criteria included adequate experience and sufficient information about the subject or phenomenon and having work experience of at least three years, the power of thinking and rethinking, the ability to express experiences, having enough time, and willingness to participate. The research environment mainly consisted of the participants’ workplaces or places where they were selected. In this study, purposive sampling continued until data saturation, because no other content or new data was added.

 To begin the interview, an electronic file containing an explanation of the interview objectives and the steps taken to design the initial conceptual framework, as well as the conceptual framework based on the results of the scoping review, was emailed to the experts. During the interview, their views on the designed conceptual framework, each of its dimensions, components, the relationships between the components, and the characteristics of each component that needed to be changed or improved were received. Next, the research team made changes to the initial conceptual framework based on the opinions of various experts and after consensus, this modified framework was sent to experts for final approval. Finally, by using the opinions of experts, the initial conceptual framework was reviewed, revised, completed, and approved.

## Results

**Figure 2:**
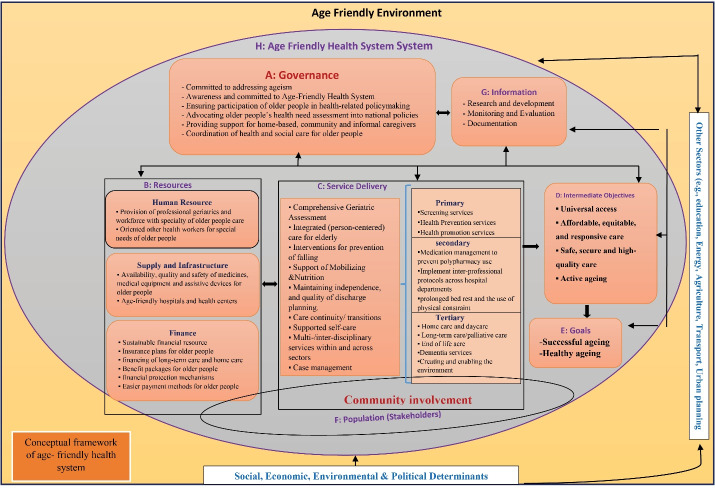
The conceptual Framework of the Age-Friendly Health System.

 In this study, after a systematic scoping review, 140 studies were included. An overview of included studies is available in the supplemental material (Supplementary file 2, Table S2). The findings of these studies were added to the basic model and after applying the opinions of experts whose demographic characteristics are presented in Table 2, the conceptual framework of the age-friendly health system was designed (Figure 2).

**Table 2:** Demographic Characteristics of the Interviewees (n = 12)

**Variables **		**No. (%)**
Gender	Male	9 (75)
	Female	3 (25)
Educational level	MSc	1 (8.3)
	PhD	11 (91.7)
Educational discipline	Healthcare management	5 (41.7)
	Specialist in health in emergencies and disaster	1 (8.3)
	Health economist	1 (8.3)
	Health policy-maker	2 (16.7)
	Geriatric Nursing	1 (8.3)
	General practitioner (geriatric MPH)	1 (8.3)
	Laboratory sciences	1 (8.3)
Total		12 (100)

Abbreviations: MSc, Master of Science; MPH, Master of Public Health; PhD, Doctor of Philosophy.

 The dimensions of this conceptual framework have specific features and the health system should function in that framework to accomplish its objectives. The stated characteristics of each dimension and component in the designed conceptual framework provide the conditions and facilities needed to provide evidence-based services and reduce the harms as a result of older people’s care so that the health system can achieve its goals regarding older people. In addition to the general characteristics of the health system, the specific characteristics of each dimension and the relationships between the components of the age-friendly health system conceptual framework can be specified as follows:

###  A: Governance

 According to findings, to strengthen age-friendly health systems that will provide acceptable, accessible, and adequate quality health services, the following factors are essential in governance and leadership sections^10,11,19-29^:

- Considering the needs of older people in designing and implementing programs, evaluating health sector programs with special emphasis on encouraging accountability, increasing justice, and performing human right-based measures on aging and health managers.
- Supporting the entry of health issues and the needs of older people into national laws, policies, and, measures related to aging, as well as national development programs.
- Increasing awareness of health and aging issues.
- Considering older people as resources for society and ensuring their participation in health and policy decisions at all levels.
- Providing a context for the engagement of older people, families, and caregivers to help healthcare providers in shared decision-making about treatment plans.

###  B: Resources

 The second dimension of the conceptual framework is recourses, which include human resources, finance, supply, and infrastructure. In each health system, providing the necessary resources and creating appropriate protocols and policies for use in different parts helps create a health system with the ability to provide special services for the elderly.^30,31^

 The human resources of an age-friendly health system are trained in appropriate relations with older people. They obtain preventive and counseling services and try to provide care for the common risk factors.^32-36^ They have improved basic knowledge and skills to provide age-friendly care.^11,21,27,28,37-43^ This includes multi-disciplinary networks of various health workforces and care facilities.^21,22,44,45^ Also, human resource strategies should be formulated in line with the needs of older people, upstream documents, the strategic position of the health system, and the broad trends of geriatric health.^46^ Employees should have continuous professional development and be allowed to enrich their knowledge and acquire new skills so as to ensure economic security. Similarly, older adults should receive high-quality and satisfactory social services.^47^

 Since older people have higher medical utilization than the general population, the health system should consider strategies for integrating and reducing healthcare costs for them.^48,49^ Older adults and other vulnerable populations are covered free-of-charge^50^ (by the National Health Insurance Fund is administered by the Ministry of Health of Bulgaria). An age-friendly health system should establish health insurance plans for older people to cover the cost of acute inpatient care, long-term management of chronic diseases, and provide access to services without financial burden^25,51-57^ and explore options to ensure adequate fiscal space for the financing of integrated and long-term care and integrated services to meet the complex needs of the elderly.^21,58-64^ The development of benefit packages for the health needs of older people and providing fair access to appropriate financial protection mechanisms for older people are some of the essential measures in this system.^21,65-67^

 Supply and infrastructure include a user-friendly physical environment for providing services that are adapted for the various physical, mental, etc needs of older people so that the physical environment and facilities of health service centers are secured to prevent the falling of older people. Also, these centers are equipped with the necessary healthcare facilities and are built and designed according to the health problems of older people.^27-29,34,40-42,51,57,68-72^ In addition, providing medical equipment and assistive devices, availability, quality, and safety of medicines and medical equipment mostly used for older people, considering polypharmacy and providing enough infrastructure such as hospital beds with exclusive older people care and daycare centers are necessary.^11,21,58,73-78^ In general, the health system’s infrastructure should be such that it can support the provision of evidence-based health services to the elderly.^79^

###  C: Service Delivery

 In this section, significant changes were made to the base model. So, every three levels of primary, secondary, and tertiary care are important and need intervention in the case of older people. Community involvement is part of the whole system. An age-friendly health system provides evidence-based care that is consistent with the full range of community-based services and significantly engages older people and their families. This system provides an opportunity for adults, families, and caregivers to act as partners and participate in joint decision-making along with healthcare providers.^10,11,24,28,37,40,41,77,80-87^ An age-friendly health system should support service delivery models that meet the health needs and expectations of older people, evaluate the effectiveness of services with a focus on age-friendliness, bridge the gaps, reduce barriers to access, and increase the quality of service according to the special health needs of older people.^25,26,42,80,88-106^ The health system will also take steps to strengthen age-friendly primary and acute health care as a suitable entry point for older people to achieve long-term continuity care. It is important to evaluate and strengthen the existing capacity to treat and manage underlying diseases; eg, through appropriate care pathways and collaboration mechanisms. Establishing and strengthening mechanisms and networks to ensure the coordinated provision of health and social care for older people with chronic conditions who need long-term care must be considered in this system.^21,31,35,77,107-132^ In this regard, in addition to emphasizing the alignment of health systems with the needs of older people, the concept of integrated care for the elderly has been introduced in the main goals and priorities set by the WHO for the decade of healthy old age. It also emphasizes the establishment of a long-term care system in each country to build understanding and commitment to the development of long-term care systems through global, regional, and local policy dialogue to bring about the necessary changes.^21,23,43,53,57,131,133-151^ Thus, the services provided should encourage the elderly to pursue physical, social, and intellectual activities that can result in an active and satisfying life.^152^

###  D and E: Outcome and Goals

 The outcomes of an age-friendly health system include better health outcomes for older adults, preventing healthcare-related avoidable harms for older people, provision of best and highest satisfied care possible for older people, enhancing the quality of care for older adults, and enabling better self-care.^11,21,22,35,53,80,153^ Finally, with the establishment of such a system, the older people community will take steps in reducing physical and cognitive disabilities caused by disease and inadequate care, which equals successful aging.^154,155^ In addition, healthy aging which is the process of maintaining the functional ability that leads to well-being at older ages^156,157^ is another goal of the age-friendly health system.

###  F: Stakeholders

 Another dimension of an age-friendly health system is stakeholders such as non-governmental organizations, the volunteer sector, families, and educational and religious organizations who are influential in the overall health system.^53,73^

###  G: Information

 In Van Olmen’s model, information was part of the resources, but in an age-friendly health system, it was transferred out of this box. Generally, it consists of research and development, monitoring and evaluation, and documentation. Effective communication- and information-sharing systems between different medical institutions, developing and implementing comprehensive aging assessment criteria, creating guidelines related to the treatment and rehabilitation of geriatric diseases, and establishing an effective referral network between different medical institutions can improve the performance of an elderly-friendly health system.^11,29,65,158^ Necessary information should be provided by various sectors of the health system for monitoring and evaluating older people’s health services by governance. In addition, needs assessment and identification of the comprehensive needs of older people are essential for proper planning and provision of appropriate services.^73^ National indicators should be designed to assess the quality of geriatric services.^92^ Necessary conditions must be provided for research to identify the needs of older people.^153^

###  H: An Age-Friendly Environment

 Another part of Van Olmen’s model is a condition which is called an age-friendly environment in an age-friendly health system. It is necessary to adjust the environment to older people’s needs. Older people need to use the environment physically and mentally through mobility and social interactions. With that respect, urban development plans should assist older adults in using urban space conveniently^26,159^ and these people mainly use urban spaces for voluntary and social activities.^160^ Since the environmental problems faced by older adults are related to various aspects of urban design, the urban landscape should be adapted to the problems of older people.^161^ An age-friendly health system can be effective if the environment is age-friendly. This environment allows everyone to take an active part in social activities, treats everyone with respect regardless of their age, and protects the most vulnerable people, helping them stay healthy and active even in senior age. Since one of the eight dimensions of the age-friendly environment is community support and health services, if the age-friendly environment is in a suitable condition in terms of the criteria related to the health system, it plays an effective role in improving the health status of the older adults and realizing the final goals of the health system.^162^

 Also, for the age-friendly health system to function well, other sectors — Education, Energy, Agriculture, Transport, and Urban planning — should be connected with this system, as well as the social, economic, environmental, and political determinants.

## Discussion

 This study aimed to design a conceptual framework for an age-friendly health system using the six stages of Arksey and O’Malley’s model of scoping reviews. Some of the dimensions of an age-friendly health system have been characterized, while there is no study with a comprehensive investigation of all its dimensions. Also, in this study, the last stage of Arksey and O’Malley’s model, which is optional, was conducted using experts in aging and health systems. This stage further improved the design of the developed conceptual framework. The proposed conceptual framework is unmatched and can function as a guide for health system managers and policymakers in the preparation to improve the quality of life and meet the needs of older people. Accordingly, the basic health system model of Van Olmen was selected. Furthermore, the basic model was modified based on the findings to determine the special characteristics of an age-friendly health system.

 In this conceptual framework, the health system is especially important, which makes it difficult to determine priority and order for its components, therefore, its dimensions are not numbered. To tackle this weakness, the numbering system was put aside and English alphabets were used instead.

 Given that old age is a period often associated with reduced physical and mental abilities and older people are regarded as vulnerable citizens, they must adapt to urban spaces. Older adults are in great need of urban spaces as well as social mobility and interactions for physical and mental reasons.^159^ They mainly use urban spaces for voluntary and social activities^160^ and urban planning projects should help them easily leave urban spaces.^159^ Since the challenges older adults face in the urban environment are related to various aspects of urban design, urban adaptation should be adjusted to the problems of older people.^161^ Therefore, an age-friendly environment must be used in this conceptual framework. Nevertheless, an age-friendly environment is absent in other health system models or conceptual frameworks, such as Frenk (1994),^163^ Londono and Frenk (1997),^164^ reforms/control knob (2004),^165^ and WHO building blocks (2007).^166^

 In this conceptual framework, the word governance was used instead of governance and leadership, because it is a comprehensive term and normally successful governance includes leadership. Some models and conceptual frameworks^167,168^ have used “management,” while management is one of the tasks of governance.

 Considering that information is a crosscutting dimension that exists throughout the system and without it, the system will not be able to perform its tasks properly. Therefore, this dimension was added to the conceptual framework and as you can see in it, its relations with other dimensions were drawn. Therefore, the information of the whole system is received by the government to be applied to monitoring, estimation of evaluation needs, and decision-making. This part has been seen in other systems, such as the WHO’s building block.^166^

 Regarding the service delivery section, community involvement is considered at all different levels of service provision (primary, secondary, and tertiary) in the conceptual framework. Given the nature of the service provided to older people, community involvement should not be ignored at any level of service delivery. In Kielmann’s model,^168^ community participation is expressed in service outcomes, input distribution, and health problems. However, in the conceptual framework of the age-friendly health system, community involvement is mentioned as an important factor in all levels of service delivery and is related to other components of the framework through service delivery.

 As we mentioned in the result section, successful aging, and healthy aging are goals of the age-friendly health system. According to Thais Abud’s study,^169^ some of the basic conditions for healthy aging are aligning health systems with the needs of older people, and developing long-term care systems^170^ which are features of the age-friendly health system. In addition, the concepts of successful aging and healthy aging are associated with longevity and the absence of disability and disease.^171,172^ Therefore, considering these conditions, healthy and successful aging will be achieved in the shadow of an age-friendly health system.

 Another important component that is important in designing an age-friendly health system’s conceptual framework is the relationship between the health system and other sectors of society such as energy, education, transportation, etc, and without coordination between them, the health system will not reach its ultimate goals. That is because an older people person is affected by the interaction between these sectors and the health sector, and the effectiveness of the measures of the health sector depends on the activities of other sectors of society.

###  Limitations

 Data synthesis was difficult due to the plety of included studies and the vastness of the investigated scopes and dimentions. Moreover, even though various articles published worldwide were examined to characterize an age-friendly health system, the lack of access to health and aging systems experts outside Iran was one of the limitations of this study. Accordingly, the opinions of these experts were not considered in the design of this conceptual framework, mainly due to the limitations imposed by COVID-19, which rendered in-person visits impossible. Finally, the interventions and measures of many countries could not be fully examined since this is a new concept, there is no set of relevant studies, and most of the existing research is in the early planning stages in many countries.

## Conclusion

 An age-friendly health system provides evidence-based care tailored to the specific needs of older people with the help of trained geriatrics and a workforce proficient in older people’s care. Governance is committed to addressing ageism and provides a context for the engagement of older people, families, and caregivers to be a part of decision-making in the treatment plan. This system focuses on both medical and psychosocial factors in older adults’ care and concentrates on maintaining their health (healthy aging), is proactive in addressing potential health needs, and improves care for those with serious illnesses who need end-of-life care. Therefore, due to the increasing population of older adults, the health system is required to prepare to meet the diverse health needs of this group in addition to performing its usual tasks, as well as dedicate the available resources to their needs and try to modify its components to in the direction of improving their quality of life.

## Ethical issues

 This paper is a part of the PhD thesis. Ethical approval was obtained at the Medical Research Ethics Committee of Tehran University of Medical Sciences (TUMS), Tehran, Iran (Ethical code: IR.TUMS.SPH.REC.1399.259).

## Competing interests

 Authors declare that they have no competing interests.

## Supplementary files

Supplementary file 1 contains Table S1.

Supplementary file 2 contains Table S2.
