# Supplementary material for: Developing a Conceptual Framework for an Age-Friendly Health System: A Scoping Review
Source: Int J Health Policy Manag. 2023 Jun 7;12:7342. doi: 10.34172/ijhpm.2023.7342 (PMC10461896; doi:10.34172/ijhpm.2023.7342)
Supplement: Supplementary file 1 — contains Table S1. [file ijhpm-12-7342-s001.pdf]

Article title: Developing a Conceptual Framework for an Age-Friendly Health System: A Scoping Review

Journal name: International Journal of Health Policy and Management (IJHPM)

Authors' information: Badrye Karami[1], Abbas Ostad-Taghizadeh[2], Arash Rashidian[1], Maryam Tajvar[1]*

[1]Department of Health Management and Economics, School of Public Health, Tehran University of Medical Sciences, Tehran, Iran.

[2]Department of Disaster & Emergency Health, School of Public Health, Tehran University of Medical Sciences, Tehran, Iran.

(*Corresponding author: Email: mtajvar@tums.ac.ir)

Citation: Karami B, Ostad-Taghizadeh A, Rashidian A, Tajvar M. Developing a conceptual framework for an agefriendly health system: a scoping review. Int J Health Policy Manag. 2023;12:7342. doi:10.34172/ijhpm.2023.7342

Supplementary file 1

Table S1. Characteristics of dimensions of an age-friendly health system, extracted from included studies

Dimensions	Specific features
**Recourses**	**Human Resource:**<br>- Trained and proficient geriatrics workforce<br>- Provision of professional geriatrics workforce with the specialty of older people care<br>-Oriented other health workers for the special needs of elderly
	**Finance:**<br>- Insurance plans for the elderly and Access to services without the financial burden<br>- Explore options to ensure adequate fiscal space for the financing of health and long-term care and integrated services<br>- Development of benefit packages for the health needs of the elderly<br>- Fair access for older people to appropriate financial protection mechanisms
	**Supply and Infrastructure:**<br>- Provide medical equipment and assistive devices<br>- Availability, Quality, and safety of medicines and medical equipment mostly used for older people<br>- Monitoring prescription and rational use of drugs preventing polypharmacy use<br>- Age-friendly hospitals and other health centers<br>- Provide enough Infrastructure including hospitals beds with the specialty of elderly care
**Service delivery**	**Common features:**

	- community involvement<br>-tailored patient-centered care<br>-care continuity/transitions<br>-Supported self-care, home-based services, person-centered care<br>-multi-/inter-disciplinary services and coordinating services within and across sectors<br>-effective communication<br>-case management<br>- reorienting the model of care<br>-Support transitions in care by implementing practices and developing partnerships that promote inter-organizational collaboration with community and post-acute services<br>- adaptation of medication management and procedures to frailty<br>-Support of mobilizing, nutrition & maintaining independence, quality of discharge planning.<br>-offer opportunities for social contact for older people, thereby enhancing social support systems and expanding social networks
	**Primary care**<br>A: **screening** (CGA)<br>1. clinical services and health assessments: Blood pressure measurement, Height, weight and body mass index measurement, Cholesterol testing, Dyslipidemia, Cardiovascular disease assessment and discussion, Diabetes, Hemoglobin, Pap smear, Mammography, Colorectal cancer assessment, Prostate cancer assessment, Vision assessment, Hearing assessment, Urinary incontinence, Depression, Annual comprehensive geriatric screening, Pneumococcal vaccination, Influenza vaccination, Tetanus vaccination, All 3 vaccinations<br>2. Health and lifestyle counselling: Calcium intake, Tobacco use/cessation, Drug and alcohol use, Healthy eating, Physical activity, Sun exposure, Oral health, Injury prevention, Poly pharmacy.<br>3. accessibility: Served by public transport, Dedicated disabled persons parking, Steps at entrance, Ramp at entrance, Entrance wider than 900 mm, Entrance accessible to wheel chair users, Emergency exits easily identifiable and accessible, Public phone near entrance, Reception counter near entrance, Room layout logical, Door widths greater than 900 mm, Seating arrangements comfortable, Floor non-slippery and well maintained, Furniture and fittings have low fall/injury risk, Availability of spare wheelchair, Corridors and rooms well-lit and ventilated, Corridors wide enough for wheelchair, Corridors free of obstructions, Hand rails or grab bars in corridors, Toilets near waiting area, Toilets wheelchair accessible, Grab bars around toilet, Fittings easy to use and accessible, Accessible shower, Eating outlet in building.<br>B: **prevention**<br>-stimulate and challenge abilities that ageing people seek to sharpen;<br>-offer the means to pursue valued interests and retain involvement in valued aspects of life<br>-facilitate participation of older people as citizens in creating responsive and inclusive local communities<br>-optimize residual abilities; which to negotiate increasing disabilities;<br>-seek to maintain a familiar environment within

	-provide support to enable management of losses as bereavement<br>-Support informal care relationships and networks.
	**Secondary care:**<br>-knowledge of attention to frailty among nurses and physicians,<br>-prolonged bed rest and the use of physical constraints,<br>-Implement inter-professional protocols across hospital departments to optimize the physical, cognitive, and psychosocial function of older patients – these processes should include high-risk screening, prevention measures, management strategies, and monitoring/evaluation processes
	**Tertiary care**<br>- Support, control and choice at the end of life<br>-Creating and enabling environment;
**Governance**	- Committed to addressing ageism<br>- Awareness and committed to Age-Friendly Health System<br>- Framing older people as resources to society and ensuring their participation in health-related policymaking at all levels.<br>- Take into account the needs of older people in the design, implementation and evaluation of health sector plans<br>- Advocate for the inclusion of health needs of older people into national laws, policies and actions on ageing<br>- Providing support for home-based, community and informal caregivers<br>- Ensuring coordinated delivery of health and social care for older people with chronic conditions and long-term care needs.
**Population (Stakeholders)**	-Groups or organizations creating or encouraging opportunities for an active and productive life and healthy lifestyle for elderly<br>- Groups or organizations denoting, building and expanding health care systems suited to older people
**Information**	- Stimulate research and development<br>- Documentation and dissemination of good practices<br>- Monitoring and Evaluation
**Outcomes**	▪ Universal access<br>▪ Acceptable, affordable, equitable, and responsive care<br>▪ Safe, secure and high-quality care<br>▪ Active ageing
**Age-friendly environment**	- Age-friendly environments foster health, well-being, and the participation of people as they age. They are accessible, equitable, inclusive, safe and secure, and supportive and promote health and prevent or delay the onset of disease and functional decline.<br>- Proving an environment for active ageing<br>- Other Sectors (e.g. education, Energy, Agriculture, Transport, Urban planning)
**Goals**	- Successful ageing<br>- Healthy ageing
