# Supplementary material for: Developing a Conceptual Framework for an Age-Friendly Health System: A Scoping Review
Source: Int J Health Policy Manag. 2023 Jun 7;12:7342. doi: 10.34172/ijhpm.2023.7342 (PMC10461896; doi:10.34172/ijhpm.2023.7342)
Supplement: Supplementary file 2 — contains Table S2. [file ijhpm-12-7342-s002.pdf]

Article title: Developing a Conceptual Framework for an Age-Friendly Health System: A Scoping Review

Journal name: International Journal of Health Policy and Management (IJHPM)

Authors' information: Badrye Karami[1], Abbas Ostad-Taghizadeh[2], Arash Rashidian[1], Maryam Tajvar[1]*

[1]Department of Health Management and Economics, School of Public Health, Tehran University of Medical Sciences, Tehran, Iran.

[2]Department of Disaster & Emergency Health, School of Public Health, Tehran University of Medical Sciences, Tehran, Iran.

(*Corresponding author: Email: mtajvar@tums.ac.ir)

Citation: Karami B, Ostad-Taghizadeh A, Rashidian A, Tajvar M. Developing a conceptual framework for an agefriendly health system: a scoping review. Int J Health Policy Manag. 2023;12:7342. doi:10.34172/ijhpm.2023.7342

Supplementary file 2

Table S2. Characteristics and summary of findings of the included studies

**Ref.<br>Langu**	**Sample, setting, design, Study place**	**Assessed dimensions of conceptual framework**	**Summary of Findings**
Wright [1],<br>2020<br>English	Cross-section study<br>Survey1: 1257 older patients who were medically complex; Survey2: 2873 older patients who visited outpatient primary care providers (PCPs).<br>The US. Idaho	Service delivery	Four essential domains of high-quality health care for older outpatients (Medications, Mobility, Mentation, and "what Matters," i.e., the 4 M's).
Winterton [2],<br>2020<br>English	Integrative review methodology<br>Rural Australian health systems	Service delivery	High-level age-friendly health-care interventions within rural Australian health systems.
Willoughby [3],<br>2020<br>English	Mixed methods study comprised Nominal group technique and a follow-up online survey.<br>10 participants based on their expertise in aged care practice, nursing, policy, research, caregiver advocacy, and quality improvement in the aged and healthcare sectors.<br>Australia	Service delivery	Developed recommendations to reduce harm and improve the quality of care for older people in Residential respite care (RRC).
Villalobos Dintrans [4],<br>2020<br>English	Review of experience of three countries (German, The Netherland, Korea) with LTC system<br>Chan School of Public Health, Boston, USA	Service delivery / stakeholders	Presents a structure to analyze long-term care systems based on four components: beneficiaries, benefits, providers, and financing.

Nora Super [5], 2020 English	Review of innovative approaches The Wharton School, University of Pennsylvania	Service delivery	Introduce new approaches for coverage of long-term care costs in older people.
Southerland [6], 2020 English	Descriptive study of 4 geriatric Emergency Department models of care The American College of Emergency Physicians	Resources	Describes developed 4 different models of comprehensive geriatric care in the Emergency Department setting.
Shaarbafchi Zadeh [7], 2020 Farsi	Descriptive-comparative approach of three countries (United States of America, Sweden and Iran) Isfahan University of Medical Sciences, Isfahan, Iran	Service delivery/ Resources	A comparison of Iran, the USA, and Sweden in terms of population health status, types of elderly services and types of elderly service providers has been.
Schwartz [8], 2020 English	Report of Half-day workshop for interprofessional trainees VA Boston Healthcare System, Boston, Massachusetts, USA	Service delivery/ Resources	Introduce the Geriatric 5Ms framework (Mobility, Mind, Medications, Multi complexity, what Matters).
Rudnicka [9], 2020 English	Descriptive study of objectives and priorities identified by WHO Department of Gynecological Endocrinology, Karowa, Warsaw, Poland	Service delivery	Introduce the main objectives and priorities identified by WHO in relation to healthy ageing.
Phua [10], 2020 English	A revive the ideals of the World Health Organization (WHO) Alma-Ata Declaration 40 years later Graduate School of Public Policy, Nazarbayev University, NurSultan, Kazakhstan.	Resources	Financing Long-term Care for All in Asia.
Pettis [11], 2020 English	A review of Age-Friendly Care for Older Adults with the NICHE Program Catholic Health Association of the United States	Resources	Alignment of NICHE Resources with Age-Friendly Health Systems Principles.
Pettis [12], 2020 English	An overview of the Age-Friendly Health Systems Initiative and a number of nurse-led programs NYU Rory Meyers College of Nursing, New York, NY, USA	Service delivery	Overview of the Age-Friendly Health Systems Initiative and how within it nurses can serve as leaders in ensuring age-friendly care for older adults.
Mudge [13], 2020 English	Interpretive phenomenological study, using open-ended interviews Royal Brisbane and Women's Hospital; University of Queensland School of Clinical Medicine. Australia	All dimensions	Barriers and enablers to achieving older person friendly hospital (OPFH) from the perspective of key informants within an academic health system.
Marsden [14], 2020 English	Descriptive qualitative study 24 semi-structured with emergency department staff and GEDI doctors and nurses. A regional hospital in Queensland, Australia	Service delivery/ Resources	To describe and evaluate the structures and processes required for the effective delivery of the e geriatric emergency department intervention (GEDI) model.
Li [15], 2020 English	A cross-sectional survey utilized mixed research methods 772 surveys with aged people (over 60 years old), 16 focus group discussions (FGDs) with 96 aged people, and 32 in-depth interviews with 16 LHWs and 16 leaders were completed in PHC sectors.	Governance	To understand the achievements made and challenges faced by healthcare management for the aged (HMA) in Southwest China.

	Department of Social Medicine and Health Service Management, Army Medical University		
Kim [16], 2020 English	Systematic review, literature review, benchmarking, focus group interviews (8 elderly inpatients, 6 family members of patients, and 14 elderly residents living in the community), and expert consultation. Konkuk University Medical Center	Service delivery	To established a senior-friendly hospital (SFH) working group for the development of a senior-specific, citizen-oriented healthcare service system.
Jung [17], 2020 English	A quasi-experimental repeated-measures design (at baseline and at 6 and 12 weeks after the intervention) Two similar LTCs for the study—they were both in Seoul	Service delivery	To develop and test the effect of the Korean Function-Focused Care Programme (K-FFCP) on cognition and physical and psychological status in long-term care facilities' (LTCs) residents.
Gori [18], 2020 English	Comment on "Financing Long-term Care: Lessons from Japan" Department of Sociology and Social Research, University of Trento, Trento, Italy	Resources	Long-term Care Financing: Politics of Long-term Care.
Gilmartin [19], 2020 English	Suggestions on how nurses can integrate assessment tools into their practice to develop individual NYU Meyers College of Nursing, United States	Service delivery	Integrating the 4Ms and the SPICES model to support age-friendly nursing care for older adults.
Fu [20], 2020 English	Design the data analysis module of the elderly health service monitoring system (HSMS) and attempt to put forward a new healthy aging (HA) model Basel, Switzerland	Goals and outcome.	To design the data analysis module of the elderly health service monitoring system (HSMS) and attempt to put forward a new healthy aging (HA) model.
Feng [21], 2020 English	Comment on "Financing Long-term Care: Lessons from Japan" RTI International, Waltham, MA, USA. The World Bank, Washington, DC, USA	Resources	Advancing Public Social Insurance for Long term Care to Meet the Global Aging Challenge.
Dintrans [22], 2020 English	Cross sectional study, propose a generic framework to assess the health system's preparedness using 3 nationally-representative sources: health status, financial protection, and responsiveness Santiago, Chile	Resources/ outcome and goal	To propose a generic framework to assess the health system's preparedness to deal with the challenges of an aging population and use it to test the Chilean health system's response to older people's health needs.
De Biasi [23], 2020 English	Original Research Article for piloting the implementation of an Age-Friendly Public Health System Florida's county health departments and at the state level, USA	Service delivery/ governance	An age-friendly public health logic model.
Casanova [24], 2020 English	Reports the findings of a comparative research focusing on the Italian and Israeli LTC systems National Institute of Health & Science on Ageing, Ancona, Italy	Service delivery	Current indicators of long-term care (LTC) demand and provision in Israel and Italy.

Cacchione [25], 2020 English	A review of past and present models of care University of Pennsylvania School of Nursing, United States	Service delivery	Review of Community based long-term care models, Long-term Care Models, Acute care.
Asadzadeh [26], 2020 Farsi	A narrative review that was conducted comparatively Iran, Tehran, University Azad Islamic, branch N	Service delivery/ information	A comparative review study to examine the care method and service provided to the elderly in Italy, Sweden, the Netherlands, Norway, Germany, Japan, Turkey and Iran.
Arakawa [27], 2020 English	Exploratory, multidisciplinary pilot study 60 participants from a geriatric Outpatient Clinic were engaged in a concurrent mixed-method approach, comprising a comprehensive geriatric survey, walking observation, semi-structured interview and an independent architectural audit. Metropolitan public hospital in Australia	Resources	Theme Analysis and Built Environment Audit: This study revealed that features of the environment can act as barriers or enablers when accessing and using a hospital.
Arain [28], 2020 English	Mixed methods approach, 76 Direct Care Staff 12 interviews with staffs for data collection Healthcare Organization in Western Canada	Service delivery	The program fits the “4 M” criteria for Elder-Friendly Care (EFC).
Allen [29], 2020 English	Describes an innovation and operations infrastructure that was successfully tested in two independent and geographically distinct community health systems Center Of Excellence in Aging and Lifelong Health, Williamsburg, Virginia.	All dimensions	An Innovation Center Model to Transform Health Systems to Improve Care of Older Adult.
Adler-Milstein [30], 2020 English	online survey of a national 797 US acute-care hospitals in 2018-2019 Acute-Care Hospitals In USA	Resources	To measure US hospitals’ adoption of electronic health record (EHR) functions focusing on structured documentation of the 4Ms and electronic health information exchange/communication with patients, caregivers, and long-term care providers.
Abbasian [31], 2020 English	Multiphasic study consists of a scoping review of existent Health care provision modalities for the elderly population worldwide, and the disposition of an optimal Health care provision scheme for the elderly population Faculty Of Health, Tabriz University of Medical Sciences, Tabriz, Iran	Service delivery	To scrutinize the best available scientific evidence on the international integrated elderly health care governance (HCG) models to revamp health care practice and policy-making processes in Iran. This model (IEHCG-IR) included four dimensions consistent with the four levels of prevention along with social support services.
Tran [32], 2019 English	Report is to review the evidence-based guidelines regarding senior friendly hospital care (A limited literature search). Canada	All dimensions	To review the evidence-based guidelines regarding senior friendly hospital care (Recommendations for Hospitals).
Ssensamba [33], 2019 English	Cross-sectional study 18 randomly selected health facilities (HFs)	Service delivery	To explored the readiness of Uganda’s public health system to offer geriatric friendly care services in Southern Central Uganda.

	Public primary health care facilities in Southern Central Uganda.		
Rahmanpour [34], 2019 Farsi	Qualitative methodological research Literature review and psychometrics validation Babol University of Medical of Sciences	Resources	Determine effective factors in an assessing tool for evaluating the structural characteristics of age friendly hospitals.
Molnar [35], 2019 English	Review of 5Ms University in Montreal, Canada	Service delivery	Geriatric 5Ms: mind, mobility, medications, Multicomplexity, and matters most.
Aseyedali [36], 2019 English	Library search method using purposeful sampling Daycare centers from Iran, Bahrain, Japan, China, UK and USA	Resources/ Service delivery	A Review on Adult Daycare Centers in the World.
Kyani [37], 2019 Farsi	Descriptive, analytic and cross-sectional study 408 physicians, nurses, and paramedical staff of hospitals from across the country by cluster sampling. Physician, nurses and paramedical staff of hospitals of Iran	Service delivery	Made items 47 age friendly hospital questionnaire.
Kuo [38], 2019 English	Observational study with a pre- and posttest design 584 employees in hospital Teaching hospital in Taiwan	Service delivery	Applying the change model to describe the AFH certification process and strategies.
Fulmer [39], 2019 English	Conceptual review of 4Ms New York University Rory Meyers College of Nursing	Service delivery	The 4Ms: Interventions and Actions.
Flaherty [40], 2019 English	Review of the Coordinating Center and Examples of the GWEP (Geriatric Workforce Enhancement Program) in Practice University of North Carolina School of Medicine (JBW), Chapel Hill, North Carolina	Resources	The Geriatric Workforce Enhancement Program (GWEP) in 2015.
Evans [41], 2019 English	Rapid scoping review of systematic reviews King's College London, Cicely Saunders Institute of Palliative Care, Policy and Rehabilitation, United Kingdom	Service delivery	Service Delivery Models to Maximize Quality of Life for Older People at the End of Life.
Bates [42], 2019 English	Research report with Semi-structured qualitative interviews with 28 practicing geriatricians, academic researchers, clinician educators, healthcare philanthropists, and representatives from professional geriatric societies. The Health Resources and Services Administration (HRSA) of the US Department of Health and Human Services (HHS)	Resources	Specific high-level interventions for the Age-Friendly Health System 4Ms.
Alimohammadzadeh [43], 2019	Qualitative methodology Interview with 30 older people over 65 years	Resources	Significant indicators from elders' viewpoints: "protective environment" and "attentive environment" of age-friendly health centers.

Farsi	Department of Health Services Management, North Tehran Branch, Islamic Azad University, Tehran, Iran		
Wankah [44], 2018 English	A qualitative multiple case study and semi-structured interviews of 28 providers Québec, Canada	Service delivery	Providers' Perspectives on the Implementation of Mandated Local Health Networks for Older People in Québec.
Vasudha [45], 2018 English	Cross sectional study, 204 patients above the age of 60 years were considered as research subjects for the study Tertiary care teaching hospital in the coastal city of Karnataka, India	Resources/ information/ stakeholders	Needs assessment by geriatrics for healthcare services.
Sivakumar [46], 2018 English	Editorial Institute of Mental Health & Neuro Sciences, Hosur Road, Bengaluru, India	Resources	Creating age friendly health systems in India.
Samir [47], 2018 English	Quasi-experimental time series analysis of 12,008 older patients admitted non-electively for acute medical issues over a 6-year period Mount Sinai Hospital, Canada	Service delivery	Mount Sinai's ACE Strategy and discusses the benefits of implementing integrated evidence-based models elder-friendly hospital interventions.
Shih [48], 2018 English	Cross sectional study 2580patients age over 65 years old and/or their companions in hospital of Taiwan Show Chwan Health Care System, Changhua County, Taiwan	Governance/ Resources	The questionnaire of Elderly Patients' Satisfaction toward Hospital Brands and Age-friendly Policies.
McCusker [49], 2018 English	Qualitative methodology. Lead ED nurses and physicians at 76 Quebec EDs: This study comprised four steps: (1) content development of the tool in collaboration with an advisory panel, (2) a survey of lead physician and nurse informants at Quebec EDs, (3) development of subscales, and (4) construct validation of the subscales. Province of Quebec, Canada	Resources	To develop and validate a comprehensive quality assessment tool for emergency department (ED) geriatric care.
Mate [50], 2018 English	Opinion paper Institute for Healthcare Improvement (IHI), United States	All dimensions	Introduce 4Ms model.
Samimi Sedeh [51], 2018 Farsi	Descriptive, analytical study 20 human resources experts The 10 high-level documentation of the health system was investigated by using Delphi technique Department of Health Services Management, Faculty of Management, Islamic Azad University, South Tehran Branch, Tehran, Iran	Resources	The Necessity of Aligning the Human Resources Strategies of Health System with the Mega-Trends of the Older People Health Domain.

Fulmer [52], 2018 English	Special article: introduce 4ms The John A. Hartford Foundation, New York, New York	Service delivery	Introduce 4Ms model.
Fulmer [53], 2018 English	Conceptual review The John A. Hartford Foundation, New York, New York	Service delivery	Introduce 4Ms model with focusing on Older Adults with Dementia.
Deschodt [54], 2018 English	Cross-sectional survey study 178 general hospitals Belgium, Denmark, Estonia, Greece, Iceland, Ireland, Malta, and Slovenia	Service delivery	Development of questionnaire according to CGA (Comprehensive geriatric assessment)-based models.
Briggs [55], 2018 English	A systematic search Department of Ageing and Life Course, World Health Organization, Geneva, Switzerland	Service delivery	The most commonly reported elements of the integrated care.
Allen [56], 2018 English	Editorial College of Medicine, Florida Atlantic University, Boca Raton, FL	Service delivery/ Resources	Introduce 4Ms model.
Tinetti [57], 2017 English	Letter to the editor Department of Medicine, Department of Public Health, Chief of Geriatrics, School of Medicine, Yale University, New Haven, CT	Service delivery	Introduce 5Ms model Mind, Mobility, Medications, Multicomplexity, and Matters.
Threapleton [58], 2017 English	A scoping review The School of Public Health and Primary Care, Prince of Wales Hospital, The Chinese University of Hong Kong, Hong Kong, China SAR	Service delivery	Integrated care for older populations and its implementation facilitators and barriers.
Pitheckoff [59], 2017 English	Conceptual review Department of Gerontology, University of Massachusetts Boston	Resources	Policies for Aging in the Republic of Bulgaria.
Pelton [60], 2018 English	Conceptual review The Institute for Healthcare Improvement, Cambridge, Massachusetts	Service delivery	More details on what the five participating health systems are learning across the 4Ms model.
World Health Organization [61], 2017 English	Report World Health Organization	All dimensions	Problems that matter for older people; For designing age friendly health system.
Lepir [62], 2017 English	Conceptual review University of Banjaluka, Faculty of political science, Department for Social work, Duke Petar Bojović boulevard 1a, Banjaluka, Bosnia and Herzegovina	Resources	Human resource management in the institutions for care of elderly people.
Kim [63], 2017	Qualitative methodology 15 participants experts	Service delivery	Development of the Korean Framework for Senior-Friendly Hospitals.

English	department of Neurology, Konkuk University Medical Center, Seoul, Korea		
Gonzalez [64], 2017 English	DH Commentary Research Faculty, Claude Pepper Center, Florida State University, Tallahassee, Florida, USA	Service delivery	A Focus on the Program of All-Inclusive Care for the Elderly (PACE).
Ghasemi [65], 2017 English	Comprehensive literature review Department of Health Education and Health Promotion, School of Public Health, Shahid Beheshti University of Medical Sciences, Tehran, Iran	Information	The most important health needs were investigated.
Fuster [66], 2017 English	Editorial Wiener Cardiovascular Institute, Icahn School of Medicine at Mount Sinai, New York, New York.	Service delivery	A New Approach to Global Health Care Due to the Aging Population.
Brouwers [67], 2017 English	Qualitative study semi-structured interviews were conducted with 18 different primary and secondary healthcare professionals working in the acute care chain Department of Public and Occupational Health, Amsterdam Public Health Research Institute, VU Medical Center, Amsterdam, the Netherlands	Service delivery	The obstacles and potential improvement opportunities of the acute care process for older patients arriving at the hospital.
Brenna [68], 2017 English	Case study an Italian region, Lombardy Department of Economics and Finance, Università Cattolica del Sacro Cuore, Milan, Italy	Resources	A Case Study of Elderly Care Financing in Lombardy, Italy.
Bastani [69], 2017 Farsi	Cross sectional study 33 hospital pharmacies Brain storming meeting with 10 geriatricians and pharmacists Hospital pharmacies in Shiraz	Resources	Evaluated the senior friendly pharmacy by research made checklist: Physical, Health care, logistics and supply services, Drug, Emotional.
Åberg [70], 2017 English	Qualitative descriptive with data being collected from focus-group interviews with 32 members of geriatric care teams Swedish university hospital with 69 beds for geriatric care	Resources	Describe factors of importance for the quality of hospital-based geriatric care from an inter-disciplinary team perspective.
Yang [71], 2016 English	A focused systematic narrative review Centre for Health Services Research Personal Social Services Research Unit, University of Kent, UK	Resources	Financing institutional long-term care for the elderly in China.
Santos [72], 2016 English	Descriptive, quantitative studying Delphi technique (the first round of the Delphi panel had 72 participants, the second 49, and the third 44 nurses with professional experience in the ES and/or researchers with publications and/or conducting research in the study area were selected)	Resources/ Service delivery	To identify and analyze the aspects necessary for an elder-friendly care in the Brazilian emergency departments from the perspective of nurses.

	Rua São Manoel, Porto Alegre, RS, Brazil		
Lu [73],<br>2016<br>English	A cross-sectional analysis using the 2010 Taiwan's Longitudinal Health Insurance Database included 999,418 beneficiaries<br>Taiwan	Resources	Comparisons of annual health care utilization, drug consumption, and medical expenditure between the elderly and general population in Taiwan.
Costley [74],<br>2016<br>English	A study exposing 13 undergraduate gerontology students to the “professional practice” of GCM in two introductory and intermediate core courses<br>Department of Health and Physical Education & Gerontological Studies and Services, York College-City University of New York, Jamaica, New York, USA	Resources	Professional Skills Through Geriatric Care Management.
Wodchis [75],<br>2015<br>English	Structured cross-case synthesis of seven integrated care programs in Australia, Canada, the Netherlands, New Zealand, Sweden, the UK and the USA.<br>Institute for Health Policy Management and Evaluation, University of Toronto; Toronto Rehabilitation Institute; Institute for Clinical Evaluative Sciences, Toronto, Canada	Service delivery	Integrating care for older people with complex needs: key insights and lessons from a seven-country cross-case analysis.
Thoma-Lürken [76],<br>2015<br>English	Descriptive cross-sectional study<br>32 long-term care organizations in the region of Limburg in The Netherlands<br>Province of Limburg/ The Netherlands	Service delivery	An Overview of Potential Labor-Saving and Quality-Improving Innovations in Long-Term Care for Older People.
McCabe [77],<br>2015<br>English	Conceptual Review<br>Department of Age-Related Health Care, Tallaght Hospital, Dublin, Ireland	Service delivery	Components of comprehensive geriatric assessment.
Kinnear [78],<br>2015<br>English	Cross-sectional analytical study<br>survey with 192 health and social care professionals across 4 NHS Trusts in England<br>College of Health and Life Sciences, Brunel University London, Uxbridge, UK	Service delivery/ Resources	Facilitators and barriers to delivering dignified care were categorized into three domains: ‘organizational level’; ‘ward level’ and ‘individual level.
Karki [79],<br>2015<br>English	Qualitative study<br>33 hospitalized older patients and 4 hospital managers in Kathmandu, Nepal<br>Department of Public Health, Nobel College, Pokhara University, Kathmandu, Nepal	Governance/ Resources/ Information	To explore the older people’s perspectives on an “elderly-friendly” hospital.
Bakker [80],<br>2015<br>English	Book (Frailty in Aging)<br>Department of Geriatric Medicine, Radboud University Medical Center, Nijmegen, The Netherlands	Service delivery	Main indicators of whether adequate care for hospitalized frail elderly patients in hospital.

Alhamdan [81], 2015 English	Descriptive cross-sectional study<br>15 randomly selected PHCCs in Riyadh City, KSA<br>Selected PHCCs in Riyadh City, KSA<br>Department of Community Health Sciences, College of Medicine, King Saud	All dimensions	To evaluate the health care services provided for older adults in PHCCs according to The World Health Organization toolkit.
Ahmadi [82], 2015 English	Descriptive study<br>26 hospitals in Tehran<br>Iran	Resources	Investigate the extent in which hospitals at Tehran meet the criteria of age-friendly hospitals (checklist with 50 items) based on the WHO age-friendly principles.
Wong [83], 2014 English	Cross Sectional Study<br>155 hospitals in Ontario<br>Every hospital in Ontario that serves adults participated in the SFH self-assessment (155 completed submissions)	Outcome and goal	To describes the application of one such framework—the Senior-Friendly Hospital (SFH) framework adopted in Ontario, SFH self-assessment survey comprised 38 questions within the five domains of the SFH framework.
Pape [84], 2014 English	Editorial<br>Department of Orthopedics/Trauma, Aachen University Medical Center, Germany	Resource	Development of geriatric trauma centers – An effort by the German Society for Trauma and Orthopedics.
Oliver [85], 2014 English	Commentaries<br>Royal Berkshire NHS Foundation Trust, Reading, UK	Service delivery	Making Health and Care Systems Fit for and Ageing Population. Why We Wrote It, Who We Wrote It For, and How Relevant It Might Be to Canada.
Kim [86], 2013 English	Descriptive study<br>191 practitioners in the medical and design fields<br>South Korea University of Florida and Mikyoung Ha, Ph.D., Yonsei University, South Korea	Resources/ Service delivery	To estimate the overall success of services at Gangnam Senior Plaza (GSP) in Korea, determine the effect of GSP on users, and ascertain local demand for services. HAPI (Healthy Body, Active Mind, Productive Lifestyle and Integrative Ageing) life model.
Daraei [87], 2014 Farsi	Review<br>Tehran, Iran	Service delivery	Evaluation of how to provide health services in private centers and NGOs in Tehran, 2014.
Woo, Jean [88], 2013 English	Qualitative methods<br>12 hospital authority staff working in the public system<br>3 randomly-selected community elderly social centers<br>Department of Medicine & Therapeutics, Faculty of Medicine, The Chinese University of Hong Kong	Service delivery	Focus group interviews among service providers for elderly people and community groups of older people using the WHO guidelines.
Tracy [89], 2013 English	Exploratory descriptive study<br>65 years of age or older patients of IMPACT clinic in the Family Practice Unit.<br>Department of Family and Community Medicine at Sunnybrook Health Sciences Centre in Toronto, Ontario	Service delivery	To design and evaluate a new interprofessional model of care for community-dwelling seniors with complex health care needs and to explore the potential of this new model as an interprofessional training opportunity.
Spoorenberg [90], 2013 English	Randomized controlled trial<br>elderly persons 75 years and older<br>Three municipalities in the province of Groningen, the Netherlands.	Service delivery	To investigate the effectiveness of Embrace, a population-based model for integrated elderly care, regarding patient outcomes, service use, costs, and quality of care.

Schwartz, Rhonda [91], 2013 English	Conceptual review Central East Regional Specialized Geriatric Services	Service delivery	SFH (senior friendly hospital) self-assessment template in Toronto, Canada.
Rechel [92], 2013 English	Report European Observatory on Health Systems and Policies, London School of Economics and Political Science, London, UK	Information	Age friendly Health systems and Ageing in the European Union.
Lee [93], 2013 English	Cross sectional study 18 operational agencies in Korea 2263 participants registered in long-term care institutions and registered in the Korean national long-term care insurance system	Service delivery	To identify the effects of community-based home healthcare projects that influence service performances in older adults.
Lakhan [94], 2013 English	Prospective cohort study 360 patients aged ≥ 70 years three metropolitan hospitals in Brisbane, Australia	Service delivery	The Higher Care at Discharge Index (HCDI): Identifying older patients at risk of requiring a higher level of care at discharge.
Kim, D [95], 2014 English	4-week survey 1188 participants aged over 50 years old South Korea	Resources	To explore the perceptions of design elements for elder-friendly hospitals: Fall prevention, Privacy, Familiarity, Wayfinding, Social support, Nature distraction, Infection prevention.
Kehusmaa [96], 2013 English	Cross sectional study 732 persons aged 65+ years Research Department, Social Insurance Institution of Finland, Helsinki, Finland	Resources	To examine to what extent informal care reduces public expenditure on elderly care.
Carpenter [97], 2013 English	Conceptual review National Center for Research Resources through the North Carolina Translational and Clinical Science Institute	Service delivery	Considerations for the geriatric-friendly Emergency Department.
World Health Organization [98], 2013 English	Meeting Report World health organization regional office for the western pacific	Service delivery/ Governance/ Resources/ Outcome and Goal	To strengthen age-friendly health systems which provide acceptable and accessible health services of sufficient quality to be effective across the care continuum.
Wideman [99], 2012 English	Conceptual review Faculty Practice and Outreach at Rush University College of Nursing and Geriatric Care, Chicago, Illinois	Service delivery	Review the (Geriatric Care Manager) GCM; The GCM is most often a nurse or social worker experienced in gerontology and case management.
Pascucci [100], 2012 English	Descriptive correlational study 52 of people aged 80 and older University of Oklahoma College of Nursing, Tulsa, Oklahoma, United States	Service delivery	To explore the factors that contribute to health promotion in the oldest old and the barriers that prevent it.
Ryan [101], 2011	Conceptual review Regional Geriatric Program of Toronto, Canada	Resources	Introduce The Senior Friendly Hospital framework.

English			
Nicholas [102], 2011 English	Conceptual review University of Rochester School of Medicine, Rochester, NY	Service delivery	Screening and Preventive Services for Older Adults.
Ontario. Waterloo Wellington [103], 2011 English	Report Regional Geriatric Program Central and Waterloo Wellington LHIN	Service delivery	Key enablers and recommendations for senior friendly care in hospitals across the WWLHIN (Waterloo Wellington LHIN).
Kelley [104], 2011 English	Qualitative study focused ethnography Interviews with seniors or their proxy decision-makers, staff and key community informants; on-site observations; a staff survey; and hospital administrative data Regional acute care hospital located in Ontario, Canada	Service delivery	Using a 'senior-friendly' conceptual framework that included the physical environment, social climate, hospital policies and procedures, and wider health care system.
Huang [105], 2011 English	Systematic Reviews/ Meta-analysis Division of Geriatric Medicine, McGill University Health Centre, Montreal, PQ, Canada	Service delivery	The Age-friendly Hospital concept; Guiding principles.
Erler [106], 2011 English	Comparative review Institute of General Practice, Johann Wolfgang Goethe-University, Frankfurt/Main, Germany	Service delivery	Preparing primary care for the future– perspectives from the Netherlands, England, and USA.
Rejeh [107], 2010 Farsi	Qualitative study Interview with 27 older people (65-81 years) Department of Nursing, Shahed University, and member of the Center for Management Research and Education of the Client - Department of Aging Health	Service delivery	Describe and explain older adults' perspectives on the experience of their hospitalization.
Rashmi [108], 2010 English	Dissertation Department of community health, john medical college Bangalore, India.	Service delivery	Make checklist 44 item for assessing senior friendly hospital include 4 domains.
Lin [109], 2010 English	Conceptual review Center for Geriatrics and Gerontology, Taipei Veterans, Taipei, Taiwan	Service delivery	Strategies of the health-care system for older people in Taipei.
Leng [110], 2010 English	Review Johns Hopkins University School of Medicine (JHU) and Peking Union Medical College (PUMC)	Service delivery/ Resources	An International Model for Geriatrics Program Development in China: The Johns Hopkins–Peking Union Medical College Experience.
Lehning [111], 2010 English	Report School of Social Welfare, University of California–Berkeley; and Bay Area Social Services Consortium, Berkeley, California, USA	Service delivery	Long-Term Care in the United States: Policy Themes and Promising Practices.

Hickman [112], 2010 English	Descriptive study School of Nursing (Sydney), Australia, The University of Notre Dame Australia	Service delivery	Describes the elements of the Chronic Care Model (CCM) and discusses how a chronic care approach can improve models of care delivery for older persons in the acute care hospital settings.
Gibson [113], 2010 English	Conceptual review Center on Aging, Department of Community Medicine, West Virginia University, Morgantown, West Virginia, U.S.A.	Service delivery	Principles of good care for long-term care facilities.
Chen [114], 2010 English	Conceptual review Institute for Geriatric Clinic and Rehabilitation, Beijing Geriatric Hospital, China.	Service delivery	Aging Beijing: Challenges and strategies of health care for the elderly.
Campbell [115], 2010 English	Comparative review University of Michigan in Ann Arbor.	Resources	Differences Between Long-Term Care (LTC) Systems, Germany and Japan, 2008
Boltz [116], 2010 English	Mixed method (Concept mapping methodology) 306 (acute care clinicians, administrators, educators, researchers, and consumers) Muriel and Virginia Pless Center at the New York University College of Nursing for Nursing Research	Service delivery	To define the core components of a system-wide acute care program designed to meet the needs of older adults.
Augustine [117], 2010 English	Dissertation Rajiv Gandhi university of health sciences, Karnataka, Bangalore, India.	Service delivery	Creating the senior friendly hospital.
Terrell [118], 2009 English	Systematic reviews and expert panel Department of Emergency Medicine (KMT), the Indiana University Center for Aging Research (KMT, DKM)	Service delivery	Quality Indicators for Geriatric Emergency Care.
Chiou [119], 2009 English	Conceptual review Institute of Public Health, National Yang-Ming University, Taiwan	Service delivery	Reviewed rationale for an age-friendly hospital (or health service) and propose our framework in detail by focusing on Taiwan's Framework of Age-Friendly Hospitals.
Boltz [120], 2008 English	Secondary analysis of data 896 registered nurses in the pre-NICHE implementation group (pre-NICHE) and 1028 nurses in the post NICHE implementation group (post-NICHE). 8 acute care hospitals in the United States	Resources	Overview of the NICHE (Nurses Improving Care for Health System Elders) tool kit: Guiding principles.
Tucker [121], 2006 English	Interventional study OASIS (Older Adult Services Inpatient Strategies) Program/Pilot Piedmont Hospital, Atlanta, Georgia.	Service delivery	OASIS (Older Adult Services Inpatient Strategies) Program/ Pilot was created to address older medical patients scattered throughout the facility and to provide geriatric support to physicians Cognition, Mobility, Depression, Vision, Hearing, and Orthostatic.
Challis [122], 2006	Cross sectional survey	Service delivery	Specialist services linked to care management.

English	130 English local authority social services departments to the 11 Trusts providing community-based Community Care Research, PSSRU, University of Manchester, Manchester, UK		
Teo [123], 2004 English	Conceptual review Department of Geography, National University of Singapore	Service delivery	Health Care for Older Persons in Singapore.
Parke [124], 2004 English	Conceptual review Older Adult Health, Chilliwack Health Services, Chilliwack, BC. Canada	Service delivery	An Elder-Friendly Hospital: translating a dream into reality.
World Health Organization [125], 2004 English	Report World Health Organization	Service delivery/ Resources,	The principles address each of the areas below: Attitudes, Training and education, Gender issues, Language, Obstructive management systems, Cost, waiting time, Appointment times that are too short for complete assessment and treatment, Lack of continuity and fragmentation of service, Special clinic or consultation hours for older persons, The physical environment.
Montero-Odasso [126], 2004 English	Conceptual review Internal Medicine Department and Geriatric Section, Hospital Italiano de Buenos Aires, Buenos Aires, Argentina	Resources/ Service delivery	Health Care for Older Persons in Argentina: A Country Profile.
Gallo [127], 2004 English	Semi experimental study 127 clinicians experienced integrated or enhanced referral care Department of Family Practice and Community Medicine, University of Pennsylvania, Philadelphia, Pa	Service delivery	Primary Care Clinicians Evaluate Integrated and Referral Models of Behavioral Health Care for Older Adults.
Crome [128], 2004 English	Conceptual review Keele University Medical School, Stoke-on-Trent, Staffordshire, UK	Service delivery	Standards of the National Service Framework for Older People, key principles and illustrative milestones.
Parks [129], 2002 English	Conceptual review Department of Family Medicine Jefferson Medical College Philadelphia, USA	Service delivery	Preventive health care for older patients.
Kodner [130], 2002 English	Conceptual review New York University, Wagner Graduate School of Public Service, New York City, New York, USA	Service delivery	The quest for integrated systems of care for frail older persons.
Coleman [131], 2002 English	Conceptual review Divisions of Geriatrics and Health Care Policy and Research, University of Colorado Health Sciences Center, Denver, Colorado, USA	Service delivery	The quest for integration in the United States, highlighting recent developments: the specific elements that comprise the continuum of health care services and are particularly relevant to meeting the needs of frail older adults.

Howe [132], 2000 English	Conceptual review Australian Association of Gerontology	Resource	Rearranging the compartments: the financing and delivery of care for Australia's elderly.
Hong [133], 2001 English	Conceptual review Department of Community, Occupational, and Family Medicine, National University of Singapore	Resource	The Savings Approach to Financing Long-Term Care in Singapore.
Godfrey [134], 2001 English	Conceptual review Nuffield Institute for Health, The University of Leeds, UK	Outcome and goal	Prevention: Developing a framework for conceptualizing and evaluating outcomes of preventive services for older people.
Parke [135], 1999 English	Conceptual review Capital Health Region in Victoria British Columbia	Service delivery	Strategies to Achieve an Elder-Friendly Hospital.
Eng [136], 1997 English	Interventional study On Lok in San Francisco, California, USA	Resources	Program of All-inclusive Care for the Elderly (PACE): an innovative model of integrated geriatric care and financing.
Kobayashi [137], 1994 English	Conceptual review Department of Public Health, Teikyo University School of Medicine, Kayo, Itabashi-ku, Tokyo Japan	Resources	Health care expenditures for the elderly and reforms in the health care system in Japan.
Kassner [138], 1992 English	Conceptual review American Association of Retired Persons	Resources	The Older Americans Act (OAA): Should Participants Share in the Cost of Services?
Berg [139], 1981 English	Conceptual review Department of Political Science. Fordham University, U.S. A	Service delivery	Examining models for coordinating health-care to the elderly.
Chiou, Shu-Ti [140], 2010 English	Conceptual review (The full article is published in: Archives of Gerontology and Geriatrics 49 Suppl. 2 (2009)Bureau of Health Promotion, Department of Health, Taiwan	Service delivery	Taiwan’s Framework of Age-friendly Hospitals and Health Services.

1. Wright MC, Fulmer T, Boult C. Preliminary Validation of a Patient-Reported Measure of the Age-Friendliness of Health Care. *Journal of the American Geriatrics Society.* 2020. doi:10.1111/jgs.16881

2. Winterton R, Hodgkin S, Clune SJ, Brasher K. Age-friendly care for older adults within rural Australian health systems: An integrative review. *Australasian Journal on Ageing.* 2020.

3. Willoughby M, Woolford MH, Young C, Ibrahim JE. Recommendations for reducing harm and improving quality of care for older people in residential respite care. *International Journal of Older People Nursing.* 2020;15(1). doi:10.1111/opn.12273

4. Villalobos Dintrans P. Designing Long-Term Care Systems: Elements to Consider. *J Aging Soc Policy.* 2020;32(1):83-99. doi:10.1080/08959420.2019.1685356
5. Super N, Burstein A, Davis J, Servat C. Innovative Strategies to Finance and Deliver Long-Term Care. *Wharton Pension Research Council Working Paper.* 2020(2020-18).
6. Southerland LT, Lo AX, Biese K, et al. Concepts in Practice: Geriatric Emergency Departments. *Annals of Emergency Medicine.* 2020;75(2):162-170. doi:https://doi.org/10.1016/j.annemergmed.2019.08.430
7. Zadeh NS, Akbari F, Khouzani PK, Zavare ASN. Comparative study of elderly care services among Iran, United States of America, and Sweden. *Scientific Journal of Kurdistan University of Medical Sciences.* 2020;25(5):123-140.
8. Schwartz AW, Hawley CE, Strong JV, et al. A Workshop for Interprofessional Trainees Using the Geriatrics 5Ms Framework. *Journal of the American Geriatrics Society.* 2020;68(8):1857-1863. doi:10.1111/jgs.16574
9. Rudnicka E, Napierała P, Podfigurna A, Męczekalski B, Smolarczyk R, Grymowicz M. The World Health Organization (WHO) approach to healthy ageing. *Maturitas.* 2020;139:6-11. doi:10.1016/j.maturitas.2020.05.018
10. Phua KH, Goh LG, Sharipova D. Ageing in Asia: Beyond the Astana Declaration Towards Financing Long-term Care for All Comment on "Financing Long-term Care: Lessons From Japan". *Int J Health Policy Manag.* 2020. doi:10.34172/ijhpm.2020.15
11. Pettis J. Achieving Age-Friendly Care for Older Adults With the NICHE Program. *JOURNAL OF THE CATHOLIC HEALTH ASSOCIATION OF THE UNITED STATES.* 2020.
12. Pettis J. Nurses leading the way to age-friendly care using the 4Ms model. *Geriatric Nursing.* 2020.
13. Mudge A, Young A, McRae P, Graham F, Whiting E, Hubbard RE. “Older people aren’t my real patients”: qualitative evaluation of barriers and enablers to older person friendly hospitals. *Authorea Preprints.* 2020.
14. Marsden EJ, Taylor A, Wallis M, et al. A structure and process evaluation of the Geriatric Emergency Department Intervention model. *Australasian Emergency Care.* 2020. doi:https://doi.org/10.1016/j.auec.2020.05.006
15. Li L, Zhang R, Chen Y, et al. Achievements and challenges in health management for aged individuals in primary health care sectors: A survey in Southwest China. *BMC Public Health.* 2020;20(1). doi:10.1186/s12889-020-8210-2
16. Kim YS, Lee J, Moon Y, et al. Development of a senior-specific, citizen-oriented healthcare service system in South Korea based on the Canadian 48/6 model of care. *BMC Geriatrics.* 2020;20(1). doi:10.1186/s12877-019-1397-3
17. Jung D, Lee H, Lee M. Function-focused care programme for older people in Korean long-term care facilities. *International Journal of Older People Nursing.* 2020;15(1). doi:10.1111/opn.12277
18. Gori C. Long-term Care Financing: Inserting Politics and Resource Allocation in the Debate Comment on "Financing Long-term Care: Lessons From Japan". *Int J Health Policy Manag.* 2020;9(2):77-79. doi:10.15171/ijhpm.2019.88
19. Gilmartin MJ. Thinking like a geriatric nurse: Integrating the 4Ms and the SPICES model to support age-friendly nursing care for older adults. *Geriatric Nursing.* 2020;41(5):662-664. doi:https://doi.org/10.1016/j.gerinurse.2020.08.014
20. Fu L, Teng T, Wang Y, He L. Data Analysis Model Design of Health Service Monitoring System for China’s Elderly Population: The Proposal of the FW Model Based on the Collaborative Governance Theory of Healthy Aging. Paper presented at: Healthcare2020.

21. Feng Z, Glinskaya E. Aiming Higher: Advancing Public Social Insurance for Longterm Care to Meet the Global Aging Challenge Comment on "Financing Long-term Care: Lessons From Japan". *Int J Health Policy Manag.* 2020;9(8):356-359. doi:10.15171/ijhpm.2019.121
22. Dintrans PV. Health systems, aging, and inequity: An example from chile. *International Journal of Environmental Research and Public Health.* 2020;17(18):1-9. doi:10.3390/ijerph17186546
23. De Biasi A, Wolfe M, Carmody J, Fulmer T, Auerbach J. Creating an age-friendly public health system. *Innovation in Aging.* 2020;4(1):igz044.
24. Casanova G, Tur-Sinai A, Lamura G. Innovating Long-Term Care Provision in Mediterranean Welfare States: A Comparison Between Italy and Israel. *Journal of Aging and Social Policy.* 2020;32(1):55-82. doi:10.1080/08959420.2019.1589888
25. Cacchione PZ. Innovative care models across settings: Providing nursing care to older adults. *Geriatric nursing (New York, N.Y.).* 2020;41(1):16-20. doi:10.1016/j.gerinurse.2020.01.011
26. Asad zadeh M, Maher A, Jaafari M, Mohammad zadeh A, Hossieni KH. Providing a model of care services for the elderly in Iran Review article (2015-2020). *Journal of Gerontology.* 2020;5(2):0-0.
27. Arakawa Martins B, Barrie H, Visvanathan R, et al. A Multidisciplinary Exploratory Approach for Investigating the Experience of Older Adults Attending Hospital Services. *HERD.* 2020:1937586720920858. doi:10.1177/1937586720920858
28. Arain MA, Graham L, Ahmad A, Cole M. Pilot implementation of elder-friendly care practices in acute care setting: A mixed methods study. *BMC Health Services Research.* 2020;20(1). doi:10.1186/s12913-020-05091-y
29. Allen K, Hazelett S, Martin M, Jensen C. An Innovation Center Model to Transform Health Systems to Improve Care of Older Adults. *Journal of the American Geriatrics Society.* 2020;68(1):15-22.
30. Adler-Milstein J, Raphael K, Bonner A, Pelton L, Fulmer T. Hospital adoption of electronic health record functions to support age-friendly care: results from a national survey. *Journal of the American Medical Informatics Association.* 2020;27(8):1206-1213.
31. Abbasian M, Shaghaghi A. An Evidence-Informed and Key Informants-Appraised Conceptual Framework for an Integrated Elderly Health Care Governance in Iran (IEHCG-IR). *Risk Manag Healthc Policy.* 2020;13:1365-1374. doi:10.2147/rmhp.s258661
32. Tran K, Wright M-D. Senior Friendly Hospital Care: A Review of Guidelines. 2019.
33. Ssensamba JT, Mukuru M, Nakafeero M, Ssenyonga R, Kiwanuka SN. Health systems readiness to provide geriatric friendly care services in Uganda: a cross-sectional study. *BMC geriatrics.* 2019;19(1):256. doi:10.1186/s12877-019-1272-2
34. Rahmanpour MS, Tirgar A, Ebadi A, Sum S, Nikpour M. Determining the components of the structural characteristics assessment tool for the age-friendly hospitals. *Journal of Gerontology.* 2019;4(1):1-10. doi:10.29252/joge.4.1.1
35. Molnar F, Frank CC. Optimizing geriatric care with the GERIATRIC 5Ms. *Canadian Family Physician.* 2019;65(1):39.
36. Masoume A, N MS, Kian TN. A Review on Adult Daycare Centers in the World *Iranian Journal of Ageing* 2019;13(4):518-529.
37. Kyani F, Farahani MMM, Hajinabi K. Factors Affecting on Development of Age_Friendly Hospital in Iran: Factor Analysis. *Journal of Clinical Nursing and Midwifery.* 2019;8(2):355-367.
38. Kuo Y-L, Chen I-J. Facilitating a change model in age-friendly hospital certification: Strategies and effects. *PloS one.* 2019;14(4):e0213496.
39. Fulmer T, Berman A. Age-Friendly Health Systems: The 4Ms. 2020.

40. Flaherty E, Busby-Whitehead J, Potter J, Lundebjerg N, Trucil DE. The Geriatric Workforce Enhancement Program: Review of the Coordinating Center and Examples of the GWEP in Practice. *The American Journal of Geriatric Psychiatry.* 2019;27(7):675-686. doi:https://doi.org/10.1016/j.jagp.2019.04.010
41. Evans CJ, Ison L, Ellis-Smith C, et al. Service Delivery Models to Maximize Quality of Life for Older People at the End of Life: A Rapid Review. *Milbank Quarterly.* 2019;97(1):113-175. doi:10.1111/1468-0009.12373
42. Bates T, Kottek A, Spetz J. Geriatrician roles and the value of geriatrics in an evolving healthcare system. *San Francisco, CA: UCSF Health Workforce Research Center on Long-Term Care.* 2019.
43. Alimohammadzadeh K, Mohebbi SF, Bolboli S, Mohebbi SM. Factors and Implications of Elder Friendly Medical Centers: A Qualitative Research. *Middle Eastern Journal of Disability Studies.* 2019;9(1):70-.
44. Wankah P, Couturier Y, Belzile L, Gagnon D, Breton M. Providers' Perspectives on the Implementation of Mandated Local Health Networks for Older People in Québec. *Int J Integr Care.* 2018;18(2):2. doi:10.5334/ijic.3098
45. Vasudha C, Dsouza B, Unnikrishnan B, Kamath R, Apuri N. Need assessment and operational feasibility indices for a geriatric inpatient facility in a tertiary care teaching hospital. *International Journal of Healthcare Management.* 2018:1-6.
46. Sivakumar P, Harbishettar V, Antony S, Thirumoorthy A. Creating age friendly health systems in India: challenges and opportunities. *Journal of Geriatric Care and Research.* 2018;5(1):1-2.
47. Sinha SK, Bennett J, Ramsden R, Bon J, Chalk T. Delivering improved patient and system outcomes for hospitalized older adults through an Acute Care for Elders Strategy. Paper presented at: Healthcare management forum2018.
48. Shih Y-J, Huang K-K, Wu H-H. Critical Factors Affecting Medical Treatment Loyalty Based on Taiwan's Framework of Age-Friendly Hospitals: A Regional Hospital Case Study of Taiwan. *Journal of Service Science Research.* 2018;10(2):233-247.
49. McCusker J, Minh Vu T, Veillette N, et al. Elder-Friendly Emergency Department: Development and Validation of a Quality Assessment Tool. *Journal of the American Geriatrics Society.* 2018;66(2):394-400.
50. Mate KS, Berman A, Laderman M, Kabcenell A, Fulmer T. Creating Age-Friendly Health Systems – A vision for better care of older adults. *Healthcare.* 2018;6(1):4-6. doi:10.1016/j.hjdsi.2017.05.005
51. GH SS, S M, S H, Sj T. The Necessity of Aligning the Human Resources Strategies of Health System with the Mega-Trends of the Older People Health Domain. *Depiction of Health http://dohweb.tbzmed.ac.ir.* 2018;9(3): 172-185(3 #p00431):-.
52. Fulmer T, Mate KS, Berman A. The age-friendly health system imperative. *Journal of the American Geriatrics Society.* 2018;66(1):22-24.
53. Fulmer T, Li N. Age-friendly health systems for older adults with dementia. *The Journal for Nurse Practitioners.* 2018;14(3):160-165.
54. Deschodt M, Boland B, Lund CM, et al. Implementation of geriatric care models in Europe (imAGE. eu): a cross-sectional survey in eight countries. *European Geriatric Medicine.* 2018;9(6):771-782.
55. Briggs AM, Valentijn PP, Thiyagarajan JA, Araujo de Carvalho I. Elements of integrated care approaches for older people: a review of reviews. *BMJ Open.* 2018;8(4):e021194. doi:10.1136/bmjopen-2017-021194
56. Allen K, Ouslander JG. Age-Friendly Health Systems: Their Time Has Come. *Journal of the American Geriatrics Society.* 2018;66(1):19-21. doi:10.1111/jgs.15134

57. Tinetti M, Huang A, Molnar F. The geriatrics 5M's: a new way of communicating what we do. *Journal of the American Geriatrics Society.* 2017;65(9):2115-2115.
58. Threapleton DE, Chung RY, Wong SYS, et al. Integrated care for older populations and its implementation facilitators and barriers: A rapid scoping review. *International Journal for Quality in Health Care.* 2017;29(3):327-334. doi:10.1093/intqhc/mzx041
59. Pitheckoff N. Aging in the Republic of Bulgaria. *Gerontologist.* 2017;57(5):809-815. doi:10.1093/geront/gnx075
60. Pelton L, Fulmer T, Hendrich A, Mate K. Creating age-friendly health systems. *Journal of the catholic health association of the united states.* 2017;32(6):62-63.
61. Organization WH. Global strategy and action plan on ageing and health. 2017.
62. Lepir L, Šćepović D, Rakanović Radonjić A. Challenges of human resource management in the institutions for care of elderly people. *IOP Conference Series: Materials Science and Engineering.* 2017;200:012030. doi:10.1088/1757-899X/200/1/012030
63. Kim Y-S, Han S-H, Hwang J-H, et al. Development of the Korean framework for senior-friendly hospitals: a Delphi study. *BMC health services research.* 2017;17(1):528.
64. Gonzalez L. A Focus on the Program of All-Inclusive Care for the Elderly (PACE). *Journal of Aging and Social Policy.* 2017;29(5):475-490. doi:10.1080/08959420.2017.1281092
65. Ghasemi S, Keshavarz Mohammadi N, Mohammadi Shahboulaghi F, Ramezankhani A. A Critical Review of Studies on Health Needs Assessment of Elderly in the World. *Elderly Health Journal.* 2017;3(1):1-9.
66. Fuster V. Changing Demographics: A New Approach to Global Health Care Due to the Aging Population. *Journal of the American College of Cardiology.* 2017;69(24):3002-3005. doi:10.1016/j.jacc.2017.05.013
67. Brouwers C, Merten H, Willems M, et al. Improving care for older patients in the acute setting: A qualitative study with healthcare providers. *Netherlands Journal of Medicine.* 2017;75(8):335-343.
68. Brenna E, Gitto L. To what extent is long-term care representative of elderly care? A case study of elderly care financing in Lombardy, Italy. *International Journal of Health Policy and Management.* 2017;6(8):467-471. doi:10.15171/ijhpm.2017.22
69. Bastani P, Dehghani M, Ahmadi Marzaleh M. Assessing Shiraz Pharmacies According to Senior-Friendly Criteria. *Salmand: Iranian Journal of Ageing.* 2017;12(1):116-123. doi:10.21859/sija-1201116
70. Åberg AC, Ehrenberg A. Inpatient geriatric care in Sweden—Important factors from an inter-disciplinary team perspective. *Archives of Gerontology and Geriatrics.* 2017;72:113-120. doi:10.1016/j.archger.2017.06.002
71. Yang W, Jingwei He A, Fang L, Mossialos E. Financing institutional long-term care for the elderly in China: a policy evaluation of new models. *Health Policy Plan.* 2016;31(10):1391-1401. doi:10.1093/heapol/czw081
72. Santos MTd, Lima MADdS, Zucatti PB. Elder-friendly emergency services in Brazil: necessary conditions for care. *Revista da Escola de Enfermagem da USP.* 2016;50(4):594-601.
73. Lu W-H, Lee W-J, Chen L-K, Hsiao F-Y. Comparisons of annual health care utilization, drug consumption, and medical expenditure between the elderly and general population in Taiwan. *Journal of Clinical Gerontology and Geriatrics.* 2016;7(2):44-47.
74. Costley AW. Exploring Skills-Based Competencies Through Geriatric Care Management Modules. *Gerontology and Geriatrics Education.* 2016;37(4):329-341. doi:10.1080/02701960.2014.990151

75. Wodchis WP, Dixon A, Anderson GM, Goodwin N. Integrating care for older people with complex needs: key insights and lessons from a seven-country cross-case analysis. *Int J Integr Care.* 2015;15:e021. doi:10.5334/ijic.2249

76. Thoma-Lürken T, Bleijlevens MHC, Lexis MAS, Hamers JPH, de Witte LP. An Overview of Potential Labor-Saving and Quality-Improving Innovations in Long-Term Care for Older People. *Journal of the American Medical Directors Association.* 2015;16(6):482-489. doi:https://doi.org/10.1016/j.jamda.2014.12.017

77. McCabe JJ, Kennelly SP. Acute care of older patients in the emergency department: strategies to improve patient outcomes. *Open access emergency medicine: OAEM.* 2015;7:45.

78. Kinnear D, Victor C, Williams V. What facilitates the delivery of dignified care to older people? A survey of health care professionals Geriatrics. *BMC Research Notes.* 2015;8(1). doi:10.1186/s13104-015-1801-9

79. Karki S, Bhatta DN, Aryal UR. Older people's perspectives on an elderly-friendly hospital environment: an exploratory study. *Risk Manag Healthc Policy.* 2015;8:81-89. doi:10.2147/rmhp.s83008

80. Bakker FC, Rikkert MGO. Hospital care for frail elderly adults: from specialized geriatric units to hospital-wide interventions. *Frailty in Aging.* Vol 41: Karger Publishers; 2015:95-106.

81. Alhamdan AA, Alshammari SA, Al-Amoud MM, et al. Evaluation of health care services provided for older adults in primary health care centers and its internal environment: A step towards age-friendly health centers. *Saudi Medical Journal.* 2015;36(9):1091-1096. doi:10.15537/smj.2015.9.11789

82. Ahmadi A, Seyedin H, Fadaye-Vatan R. Towards age-friendly hospitals in developing countries: a case study in Iran. *Health promotion perspectives.* 2015;5(1):42.

83. Wong KS, Ryan DP, Liu BA. A system-wide analysis using a senior-friendly hospital framework identifies current practices and opportunities for improvement in the care of hospitalized older adults. *Journal of the American Geriatrics Society.* 2014;62(11):2163-2170.

84. Pape HC, Friess T, Liener U, et al. Development of geriatric trauma centers-An effort by the German Society for Trauma and Orthopaedics. *Injury.* 2014;45(10):1513-1515. doi:10.1016/j.injury.2014.08.006

85. Oliver D. Re: making health and care systems fit for and ageing population. Why we wrote it, who we wrote it for, and how relevant it might be to Canada. *Can Geriatr J.* 2014;17(4):136-139. doi:10.5770/cgj.17.182

86. Kim J. 'HAPI' life model for the new older generation in Korea. *Australas J Ageing.* 2013;32(2):135-141. doi:10.1111/ajag.12082

87. Daraei Z, Maher A, Ali Mohammadzade K. Evaluation of how to provide health services in private centers and NGOs in Tehran. 2014.

88. Woo J, Mak B, Yeung F. Age-friendly primary health care: An assessment of current service provision for older adults in Hong Kong. *Health services insights.* 2013;6:HSI. S12434.

89. Tracy CS, Bell SH, Nickell LA, Charles J, Upshur REG. The IMPACT clinic: Innovative model of interprofessional primary care for elderly patients with complex health care needs. *Canadian Family Physician.* 2013;59(3):e148-e155.

90. Spoorenberg SLW, Uittenbroek RJ, Middel B, Kremer BPH, Reijneveld SA, Wynia K. Embrace, a model for integrated elderly care: Study protocol of a randomized controlled trial on the effectiveness regarding patient outcomes, service use, costs, and quality of care. *BMC Geriatrics.* 2013;13(1). doi:10.1186/1471-2318-13-62

91. Wong K, Tsang A, Liu B. Senior Friendly Hospital Care in the Central East Local Health Integration Network; Summary of Self-Assessment Responses. Regional Geriatric Program of Toronto: Local health integration network 2015.

92. Rechel B, Grundy E, Robine J-M, et al. Ageing in the European Union. *The Lancet.* 2013;381(9874):1312-1322. doi:https://doi.org/10.1016/S0140-6736(12)62087-X

93. Lee T. Community-Based Home Healthcare Project for Korean Older Adults. *Osong Public Health and Research Perspectives.* 2013;4(5):233-239. doi:https://doi.org/10.1016/j.phrp.2013.09.002

94. Lakhan P, Jones M, Wilson A, Gray LC. The Higher Care At Discharge Index (HCDI): Identifying older patients at risk of requiring a higher level of care at discharge. *Archives of Gerontology and Geriatrics.* 2013;57(2):184-191. doi:10.1016/j.archger.2013.04.003

95. Kim D, Lee JH, Ha M. Exploring Perceptions of Designers and Medical Staff in South Korea about Design Elements for the Elder-Friendly Hospital. *Journal of Interior Design.* 2014;39(4):15-32.

96. Kehusmaa S, Autti-Rämö I, Helenius H, Rissanen P. Does informal care reduce public care expenditure on elderly care? Estimates based on Finland's Age Study. *BMC Health Services Research.* 2013;13(1). doi:10.1186/1472-6963-13-317

97. Carpenter CR, Platts-Mills TF. Evolving prehospital, emergency department, and “inpatient” management models for geriatric emergencies. *Clinics in geriatric medicine.* 2013;29(1):31-47.

98. (WHO) WHO. *Meeting on Ageing and Health in the Western Pacific, Manila, Philippines, 09-11 July 2013: report*: Manila: WHO Regional Office for the Western Pacific;2013.

99. Wideman M. Geriatric care management: Role, need, and benefits. *Home Healthcare Nurse.* 2012;30(9):553-559. doi:10.1097/NHH.0b013e31822a0637

100. Pascucci MA, Chu N, Renee Leasure A. Health promotion for the oldest of old people. *Nursing Older People.* 2012;24(3):22-28. doi:10.7748/nop2012.04.24.3.22.c9017

101. Ryan D, Liu B, Awad M, Wong K. Improving older patients’ experience in the emergency room: the senior-friendly emergency room. *Aging Health.* 2011;7(6):901-909.

102. Nicholas JA, Hall WJ. Screening and preventive services for older adults. *Mount Sinai Journal of Medicine.* 2011;78(4):498-508. doi:10.1002/msj.20275

103. Network OWWLHI, Smith CS, Noor A, Sarkaria G. *A Summary of Senior Friendly Care in Waterloo Wellington Local Health Integration Network Hospitals*: Waterloo Wellington Local Health Integration Network; 2011.

104. Kelley ML, Parke B, Jokinen N, Stones M, Renaud D. Senior-friendly emergency department care: an environmental assessment. *Journal of Health Services Research & Policy.* 2011;16(1):6-12. doi:10.1258/jhsrp.2010.009132

105. Huang AR, Larente N, Morais JA. Moving towards the age-friendly hospital: A paradigm shift for the hospital-based care of the elderly. *Canadian geriatrics journal: CGJ.* 2011;14(4):100.

106. Erler A, Bodenheimer T, Baker R, et al. Preparing primary care for the future - perspectives from the Netherlands, England, and USA. *Z Evid Fortbild Qual Gesundhwes.* 2011;105(8):571-580. doi:10.1016/j.zefq.2011.09.029

107. Rejeh N, Heravi-Karimooi M, Foroughan M. The Needs of Hospitalized Elderly Patients: A Qualitative Study. *Salmand: Iranian Journal of Ageing.* 2010;5(1):0-0.

108. Rashmi M. *Senior friendly hospitals in Bangalore city-development And application of criteria*2010.
109. Lin MH, Chou MY, Liang CK, Peng LN, Chen LK. Population aging and its impacts: Strategies of the health-care system in Taipei. *Ageing Research Reviews.* 2010;9(SUPPL.):S23-S27. doi:10.1016/j.arr.2010.07.004
110. Leng SX, Tian X, Liu X, et al. An international model for geriatrics program development in China: the Johns Hopkins-Peking Union Medical College experience. *J Am Geriatr Soc.* 2010;58(7):1376-1381. doi:10.1111/j.1532-5415.2010.02927.x
111. Lehning AJ, Austin MJ. Long-Term Care in the United States: Policy Themes and Promising Practices. *Journal of Gerontological Social Work.* 2010;53(1):43-63. doi:10.1080/01634370903361979
112. Hickman LD, Rolley JX, Davidson PM. Can principles of the Chronic Care Model be used to improve care of the older person in the acute care sector? *Collegian.* 2010;17(2):63-69. doi:10.1016/j.colegn.2010.05.004
113. Gibson MC, Carter MW, Helmes E, Edberg AK. Principles of good care for long-term care facilities. *International Psychogeriatrics.* 2010;22(7):1072-1083. doi:10.1017/S1041610210000852
114. Chen Z, Yu J, Song Y, Chui D. Aging beijing: Challenges and strategies of health care for the elderly. *Ageing Research Reviews.* 2010;9(SUPPL.):S2-S5. doi:10.1016/j.arr.2010.07.001
115. Campbell JC, Ikegami N, Gibson MJ. Lessons from public long-term care insurance in Germany and Japan. *Health Affairs.* 2010;29(1):87-95. doi:10.1377/hlthaff.2009.0548
116. Boltz M, Capezuti E, Shabbat N. Building a framework for a geriatric acute care model. *Leadership in Health Services.* 2010.
117. Augustine V. *Senior friendly hospital strategy; Needs and Challenges,-A study in a selected hospital*, RGUHS; 2010.
118. Terrell KM, Hustey FM, Hwang U, Gerson LW, Wenger NS, Miller DK. Quality indicators for geriatric emergency care. *Academic Emergency Medicine.* 2009;16(5):441-449. doi:10.1111/j.1553-2712.2009.00382.x
119. Chiou S-T, Chen L-K. Towards age-friendly hospitals and health services. *Archives of gerontology and geriatrics.* 2009;49:S3-S6.
120. Boltz M, Capezuti E, Bowar-Ferres S, et al. Changes in the geriatric care environment associated with NICHE (Nurses Improving Care for HealthSystem Elders). *Geriatric Nursing.* 2008;29(3):176-185.
121. Tucker D, Bechtel G, Quartana C, et al. The OASIS program: redesigning hospital care for older adults. *Geriatric nursing.* 2006;27(2):112-117.
122. Challis D, Stewart K, Donnelly M, Weiner K, Hughes J. Care management for older people: Does integration make a difference? *Journal of Interprofessional Care.* 2006;20(4):335-348. doi:10.1080/13561820600727130
123. Teo P. Health care for older persons in Singapore: Integrating state and community provisions with individual support. *Journal of Aging and Social Policy.* 2004;16(1):43-67. doi:10.1300/J031v16n01_03
124. Parke B, Brand P. An Elder-Friendly Hospital: translating a dream into reality. *Canadian journal of nursing leadership.* 2004;17(1):62-76.
125. Organization WH. *Towards age-friendly primary health care*: World Health Organization; 2004.
126. Montero-Odasso M, Przygoda P, Redondo N, Adamson J, Kaplan R. Health care for older persons in Argentina: A country profile. *Journal of the American Geriatrics Society.* 2004;52(10):1761-1765. doi:10.1111/j.1532-5415.2004.52474.x
127. Gallo JJ, Zubritsky C, Maxwell J, et al. Primary care clinicians evaluate integrated and referral models of behavioral health care for older adults: Results from a multisite effectiveness trial (PRISM-E). *Annals of Family Medicine.* 2004;2(4):305-309. doi:10.1370/afm.116

128. Crome P, Natarajan I. The national service framework for older people: England's approach to ending age discrimination in services and therapeutics. *Drugs and Aging.* 2004;21(8):499-510. doi:10.2165/00002512-200421080-00002
129. Parks SM, Hsieh C. Preventive health care for older patients. *Primary Care - Clinics in Office Practice.* 2002;29(3):599-614. doi:10.1016/S0095-4543(02)00005-2
130. Kodner DL. The quest for integrated systems of care for frail older persons. *Aging Clinical and Experimental Research.* 2002;14(4):307-313. doi:10.1007/BF03324455
131. Coleman EA. Challenges of systems of care for frail older persons: The United States of America experience. *Aging Clinical and Experimental Research.* 2002;14(4):233-238. doi:10.1007/bf03324444
132. Howe AL. Recent developments in aged care policy in Australia. *Journal of Aging and Social Policy.* 2001;13(2-3):101-116. doi:10.1300/J031v13n02_08
133. Hong PK. The savings approach to financing long-term care in Singapore. *Journal of Aging and Social Policy.* 2001;13(2-3):169-183. doi:10.1300/J031v13n02_12
134. Godfrey M. Prevention: Developing a framework for conceptualizing and evaluating outcomes of preventive services for older people. *Health and Social Care in the Community.* 2001;9(2):89-99. doi:10.1046/j.1365-2524.2001.00283.x
135. Parke B, Stevenson L. Creating an Elder-Friendly Hospital. *Healthcare Management Forum.* 1999;12(3):45-48. doi:https://doi.org/10.1016/S0840-4704(10)60717-X
136. Eng C, Pedulla J, Eleazer GP, McCann R, Fox N. Program of All-inclusive Care for the Elderly (PACE): an innovative model of integrated geriatric care and financing. *J Am Geriatr Soc.* 1997;45(2):223-232. doi:10.1111/j.1532-5415.1997.tb04513.x
137. Kobayashi Y. Health care expenditures for the elderly and reforms in the health care system in Japan. *Health Policy.* 1994;29(3):197-208. doi:https://doi.org/10.1016/0168-8510(94)90051-5
138. Kassner E. The Older Americans Act: should participants share in the cost of services? *J Aging Soc Policy.* 1992;4(1-2):51-71. doi:10.1300/J031v04n01_06
139. Berg BF. Examining models for coordinating health-care to the elderly. *Computers, Environment and Urban Systems.* 1981;6(2):97-109.
140. Chiou S-T. Taiwan’s Framework of Age-friendly Hospitals and Health Services.
